# Self-adjusting binding pockets enhance H_2_ and CH_4_ adsorption in a uranium-based metal–organic framework†
†Electronic supplementary information (ESI) available: Synthetic, analytical and crystallographic details. Single crystal X-ray crystallographic data was deposited in the Cambridge Crystallographic Data Centre database. CCDC 1996337. For ESI and crystallographic data in CIF or other electronic format see DOI: 10.1039/d0sc02394a


**DOI:** 10.1039/d0sc02394a

**Published:** 2020-05-27

**Authors:** Dominik P. Halter, Ryan A. Klein, Michael A. Boreen, Benjamin A. Trump, Craig M. Brown, Jeffrey R. Long

**Affiliations:** a Department of Chemistry , University of California , Berkeley , CA 94720 , USA . Email: jrlong@berkeley.edu; b Materials Sciences Division , Lawrence Berkeley National Laboratory , Berkeley , CA 94720 , USA; c Chemistry and Nanoscience Department , National Renewable Energy Laboratory , Golden , CO 80401 , USA; d Center for Neutron Research , National Institute of Standards and Technology , Gaithersburg , MD 20899 , USA; e Chemical Sciences Division , Lawrence Berkeley National Laboratory , Berkeley , CA 94720 , USA; f Department of Chemical Engineering , University of Delaware , Newark , DE 19716 , USA; g Department of Chemical and Biomolecular Engineering , University of California , Berkeley , CA 94720 , USA

## Abstract

A new, air-stable, permanently porous uranium(iv) metal–organic framework U(bdc)_2_ (**1**, bdc^2–^ = 1,4-benzenedicarboxylate) was synthesized and its H_2_ and CH_4_ adsorption properties were investigated. Low temperature adsorption isotherms confirm strong adsorption of both gases in the framework at low pressures. *In situ* gas-dosed neutron diffraction experiments with different D_2_ loadings revealed a rare example of cooperative framework contraction (Δ*V* = –7.8%), triggered by D_2_ adsorption at low pressures. This deformation creates two optimized binding pockets for hydrogen (*Q*_st_ = –8.6 kJ mol^–1^) per pore, in agreement with H_2_ adsorption data. Analogous experiments with CD_4_ (*Q*_st_ = –24.8 kJ mol^–1^) and *N*,*N*-dimethylformamide as guests revealed that the binding pockets in **1** adjust by selective framework contractions that are unique for each adsorbent, augmenting individual host–guest interactions. Our results suggest that the strategic combination of binding pockets and structural flexibility in metal–organic frameworks holds great potential for the development of new adsorbents with an enhanced substrate affinity.

## Abstract 2

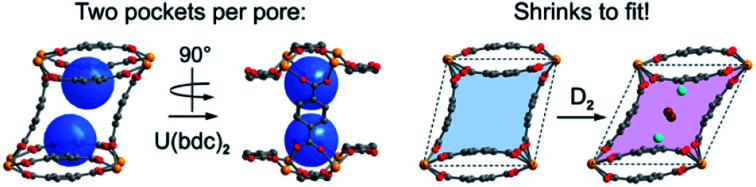
Optimizing binding pocket geometries in MOFs for ideal interaction with target molecules remains a tremendous synthetic challenge. In the new MOF U(bdc)_2_, self-adjusting binding pockets flex to bind differently sized guests H_2_, CH_4_ and DMF.

## Introduction

Metal–organic frameworks are a class of chemically-robust, porous, and often rigid materials, composed of metal ions or clusters connected by bridging organic linkers.^1^–^4^ The physical and chemical properties of these materials are highly tunable based on choice of metal and linker, and thus metal–organic frameworks have been proposed for a wealth of applications,^5^–^9^ including catalysis,^10^–^15^ sensing,^16^–^18^ carbon capture,^19^–^23^ gas separations,^24^–^26^ and gas storage.^27^–^31^ Metal–organic frameworks have attracted particular interest as candidate gas storage materials for H_2_ and CH_4_ that could enable more efficient use of these energy carriers as cleaner fuel alternatives.^32^–^38^ However, significant advances are still needed to develop frameworks capable of maintaining interactions with these guests at ambient temperatures.^39^–^41^

Two main strategies have been developed to achieve strong binding of H_2_ and CH_4_ in metal–organic frameworks. The first approach utilizes materials with coordinatively-unsaturated metal sites, which can polarize and strongly bind various guests.^42^,^43^ Representative of this materials class is the framework Ni_2_(*m*-dobdc) (*m*-dobdc^4–^ = 4,6-dioxido-1,3-benzenedicarboxylate), which is currently the top performing material for ambient temperature, physisorptive H_2_ storage.^33^,^34^ The other strategy exploits tight binding pockets in small-pore frameworks, which can engage in multiple, weak interactions with guest molecules to achieve strong overall guest binding, analogous to shape-selective molecular recognition in enzymes.^44^ An example of how such cumulative dispersion forces can outperform strong interactions at open metal sites is the adsorption of CH_4_ in Cu_2_(btc)_3_ (HKUST-1, btc^3–^ = 1,3,5-benzenetricarboxylate).^45^ This material exhibits open metal sites and binding pockets in direct competition for CH_4_ adsorption. Structural characterization of Cu_2_(btc)_3_ dosed with low pressures of CD_4_ confirmed that methane preferably adsorbs at the binding pockets inside small octahedral cages of the framework, rather than through direct interactions at the copper(ii) open metal sites. The reason for this behavior is that the multiple interactions inside the pore give rise to a higher overall binding energy than that achieved with a CH_4_ molecule adsorbed at a single copper(ii) center (–21.8 *versus* –9.4 kJ mol^–1^, respectively).^45^

Cumulative dispersion interactions between guest molecules and framework pockets decrease exponentially with the adsorbate–framework distances (*F* ∝ 1/*r*^6^), and therefore require a precise geometric fit between guest and binding pocket.^46^ For example, as a result of its smaller kinetic diameter relative to CH_4_,^47^ H_2_ preferentially binds at the open metal sites of Cu_2_(btc)_3_, rather than in the hexagonal pockets.^48^ The development of new frameworks with efficient binding pockets therefore requires precise optimization for each adsorbate of interest, although achieving this goal by structural design remains a significant challenge.

An alternative approach to circumvent the synthetic intricacy of developing materials with optimized guest–specific binding pockets, are materials that combine small binding pockets with moderate framework flexibility.^49^ Synthetic tuning can thus be used to design crude binding pockets, which are capable of self-adjusting in response to guest adsorption. Together, these design features could enable access to optimal binding pocket geometries for a variety of guests within the same material. Such molecular recognition often relies on initially weak host–guest interactions, highlighting the importance to precisely adjust the energy required for the deformation of a flexible framework and the energy released by guest adsorption.^50^–^52^

Flexibility is typically introduced into metal–organic frameworks by utilizing organic linkers with non-rigid stems, by interconnecting metals with non-chelating linkers, or by cross-linking two-dimensional frameworks with additional ditopic but weakly binding linkers.^53^–^55^ Prominent examples are M(OH)(bdc) (MIL-53; bdc^2–^ = 1,4-benzenedicarboxylate; M = Fe, Cr, Sc, Al, or Ga)^56^–^60^ and M_3_(O)(OH)(H_2_O)_2_(bdc)_3_ (MIL-88; M = Fe, Cr).^61^ These frameworks undergo drastic geometric distortions upon guest adsorption, often referred to as framework swelling, which can induce a substantial unit cell volume increase of up to 74%, as shown for example by CO_2_ adsorption in Fe(OH)(bdc).^62^ Such large structural changes are too extreme to drive the subtle binding pocket adjustments sought here. One could instead envision limiting the flexibility of non-chelating bdc^2–^ linkers by substantially increasing the number of metal–ligand bonds per metal node. A higher coordination number should limit structural rearrangements by causing steric encumbrance around the metal nodes and increase rigidity by further crosslinking the resulting material. Additionally, a higher ligand-to-metal ratio could result in smaller pore sizes and better binding pockets.

With their tendency to adopt high coordination numbers, actinides are well suited as metal nodes for the development of such materials.^63^ We chose depleted uranium to test our hypothesis, as it is only mildly radioactive and because a limited but growing number of uranium-based frameworks have already been reported and could guide the synthesis.^64^ Inspired by previous work on the synthesis of porous metal–organic frameworks from uranium(iv) and bdc^2–^ linkers,^65^ we synthesized a new, three-dimensional U(bdc)_2_ phase (**1**) with permanent porosity and a moderate level of structural flexibility. Using a combination of gas adsorption studies and *in situ* powder neutron diffraction experiments, we demonstrate that this framework undergoes an adjustable contraction of its pores to accommodate and strongly bind H_2_ and CH_4_, with different levels of contraction and host–guest interactions for each molecule.

## Results and discussion

**Fig. 1:**
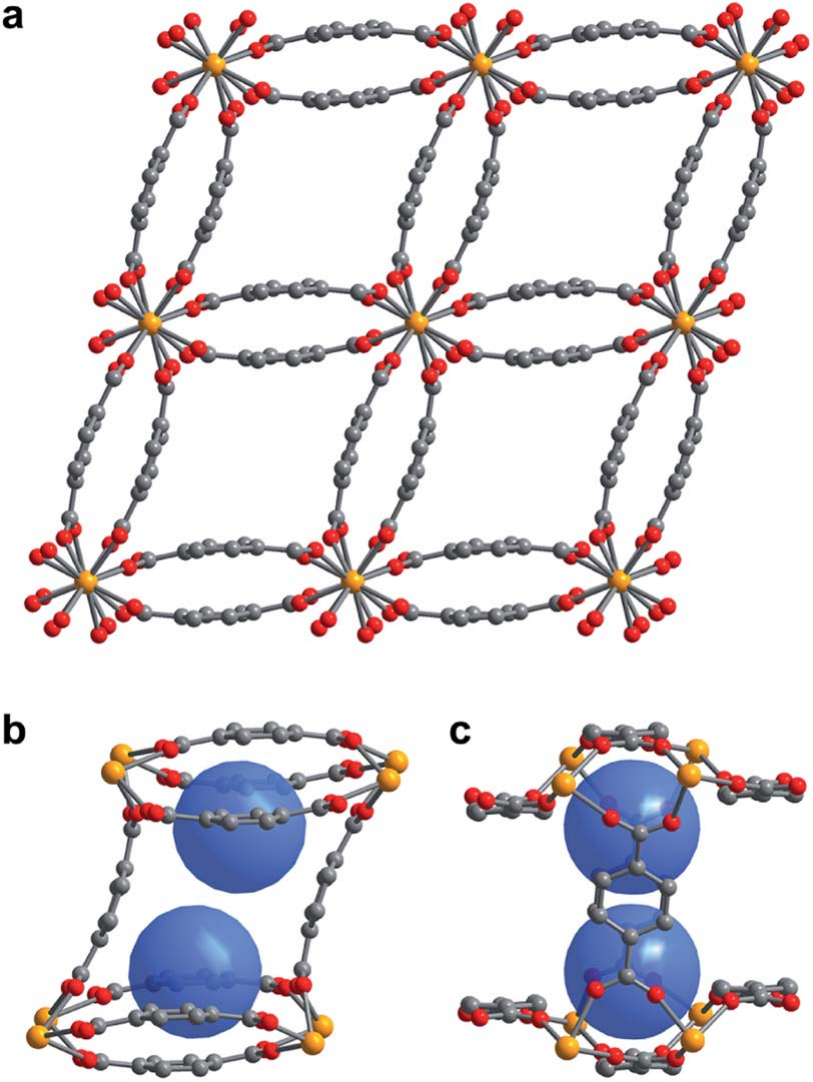
(a) Single crystal X-ray diffraction structure of **1–H_2_O** viewed along the *c*-axis, showing the parallelepipedal pores. (b) Truncated structure showing one of the pores of **1–H_2_O** along the crystallographic *c*-axis, with the two identical binding pockets of the pore depicted as blue spheres. (c) The same view as in (b), rotated by 90° to visualize the bowl-shaped arrangement of three bdc^2–^ linkers that form the cap of each binding pocket. Orange, red, and grey spheres represent U, O, and C atoms, respectively; solvent and H atoms are omitted for clarity.

The compound U(bdc)_2_·4H_2_O (**1–H_2_O**) was synthesized through the reaction of UI_4_(1,4-dioxane)_2_ with H_2_bdc in *N*,*N*-dimethylformamide (DMF, <0.15% water content as received) at 140 °C under argon inside a Parr autoclave. After three days, the material was isolated in 79% yield as air-stable, thin emerald green needle-shaped crystals. Single crystal X-ray diffraction analysis was used to determine the structure of **1–H_2_O** (Fig. 1), and selected bond distances and angles are given in Table S5 of the ESI.† We note that powder X-ray diffraction patterns collected for bulk samples of **1–H_2_O** match the simulated pattern determined from single-crystal data, confirming the bulk purity of the crystalline material (see ESI, Fig. S7†).

Compound **1–H_2_O** crystallizes in the space group *C*2/*c* and features eight-coordinate uranium centers in a distorted square-antiprismatic environment. Each uranium(iv) is coordinated to one oxygen atom of eight different bdc^2–^ linkers, and all linkers are coordinated to four different uranium ions in a bridging fashion. This motif results in an overall framework structure consisting of distorted parallelepipedal pores (Fig. 1a) formed by chains of uranium(iv) centers that propagate along the crystallographic *c*-axis (see ESI, Fig. S17†) and are bridged by bdc^2–^ linkers bent in a concave and convex fashion.

Each pore is formed by two opposing, inwardly bent bdc^2–^ linkers at the sides and is capped at the top and bottom by a bowl-shaped arrangement of three additional bdc^2–^ linkers (Fig. 1b and c). The resulting geometry yields two identical binding pockets per pore that are ∼5 Å in diameter and related by an inversion center.

In the as-synthesized framework, each binding pocket is occupied by two disordered water molecules, yielding the composition U(bdc)_2_·4H_2_O, which was also confirmed by thermogravimetric analysis (see ESI, Fig. S6†). While disorder precluded modeling of any specific interactions, the guest water molecules are likely to engage in hydrogen bonding with each other and with the highly polarized U–O bonds. We note that the structure of the pores is such that guests could engage in a variety of additional interactions, including with the linker π-systems and arene C–H bonds.

Activated U(bdc)_2_ (**1**) was obtained by heating **1–H_2_O** at 260 °C for 10 h under dynamic vacuum. Combustion analysis confirmed the empirical formula for **1** and the removal of guest water molecules. Nitrogen adsorption isotherms obtained at 77 K for four different samples revealed the activated material is permanently porous, with an average Langmuir surface area of 497 ± 6 m^2^ g^–1^ (see ESI, Fig. S1†). Powder X-ray diffraction analysis confirmed that **1** remains crystalline with a slightly different structure from that of its solvated analogue (see ESI, Fig. S7†). Interestingly, while many flexible frameworks contract or even fully collapse to a nonporous structure upon solvent removal,^37^,^66^–^68^ activation of **1–H_2_O** to give **1** results in an expansion of the framework along the crystallographic *b*-axis, from 12.598(1) to 12.812(1) Å. This change results in an increase in the unit cell volume from 1887.4(3) to 1902.1(2) Å^3^, while retaining the *C*2/*c* space group. This behavior upon guest removal indicates that the framework binding pockets are indeed able to contract to improve interactions with adsorbates.

**Fig. 2:**
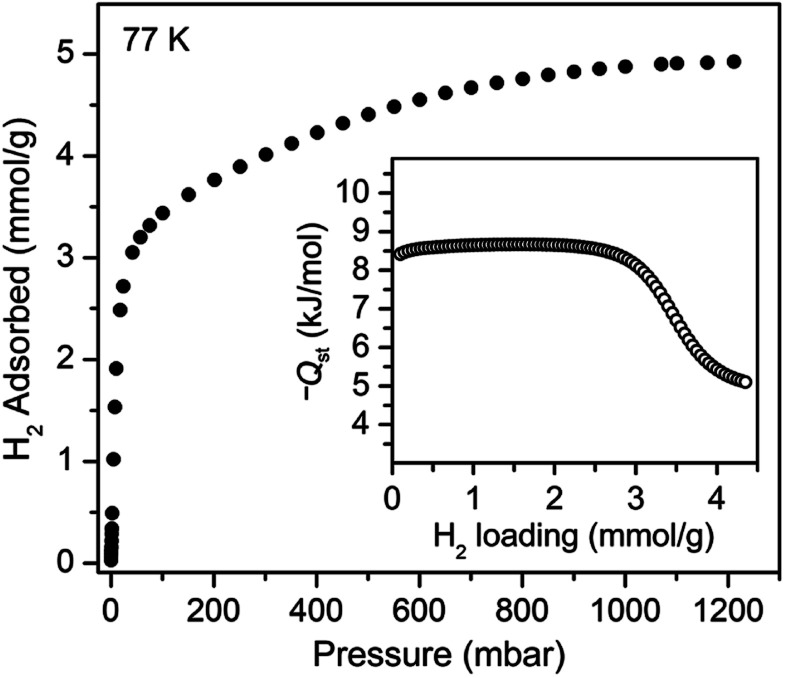
Hydrogen adsorption isotherm for **1**, measured at 77 K. Inset: loading-dependent isosteric heat of adsorption (*Q*_st_) for H_2_ in **1**.

We sought to study the flexibility of **1** in more detail using H_2_ and CH_4_ (with kinetic diameters of 2.9 and 3.8 Å, respectively)^18^ as probe molecules of interest for potential gas storage applications. The low-pressure H_2_ adsorption isotherm for U(bdc)_2_ at 77 K exhibits an initial steep rise to ∼3.5 mmol g^–1^ at 115 mbar, which is indicative of the presence of strong adsorption sites (Fig. 2). We note that this loading corresponds to the theoretical capacity expected for adsorption of one H_2_ molecule per adsorption pocket (two per pore). With further increasing pressure, the quantity of adsorbed H_2_ increases very gradually to an apparent saturation value of ∼4.9 mmol g^–1^ at 1.2 bar. A dual site Langmuir model was used to fit independently H_2_ adsorption data collected at 77 and 87 K (see Section 3 of the ESI, Fig. S2 and Table S1†), and the Clausius–Clapeyron equation was then employed to calculate the isosteric heat (*Q*_st_) of H_2_ adsorption as a function of loading (Fig. 2, inset). For loadings up to 2.5 mmol g^–1^, H_2_ adsorbs exclusively at primary binding sites in the framework pockets (see below) to give a *Q*_st_ of –8.6 kJ mol^–1^. Notably, this value is larger in magnitude than the H_2_ isosteric heat of adsorption in activated carbon materials (–5.0 to –6.4 kJ mol^–1^)^69^ and the majority of frameworks with coordinatively-saturated metal sites (–4.1 to –8.8 kJ mol^–1^).^70^

**Fig. 3:**
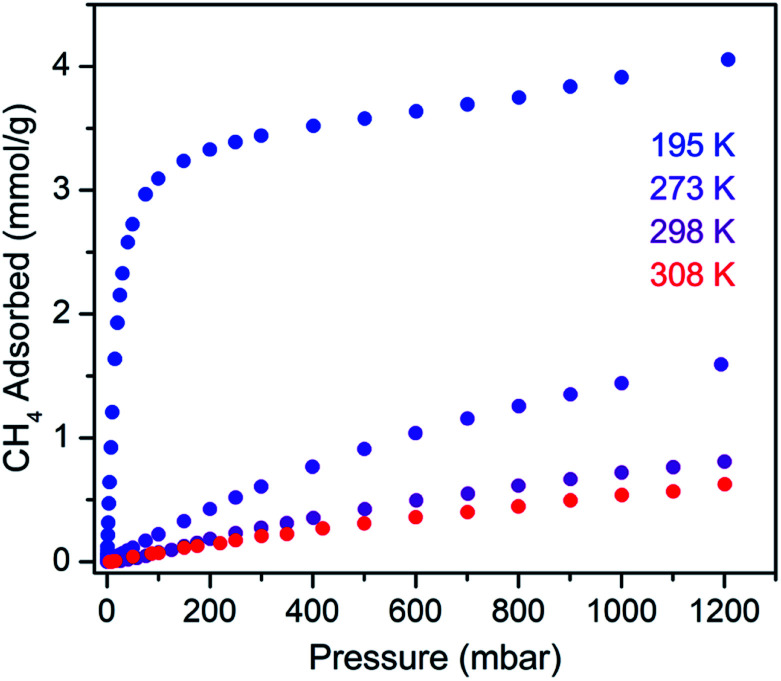
Methane adsorption isotherms for **1**, measured at the indicated temperatures.

**Fig. 4:**
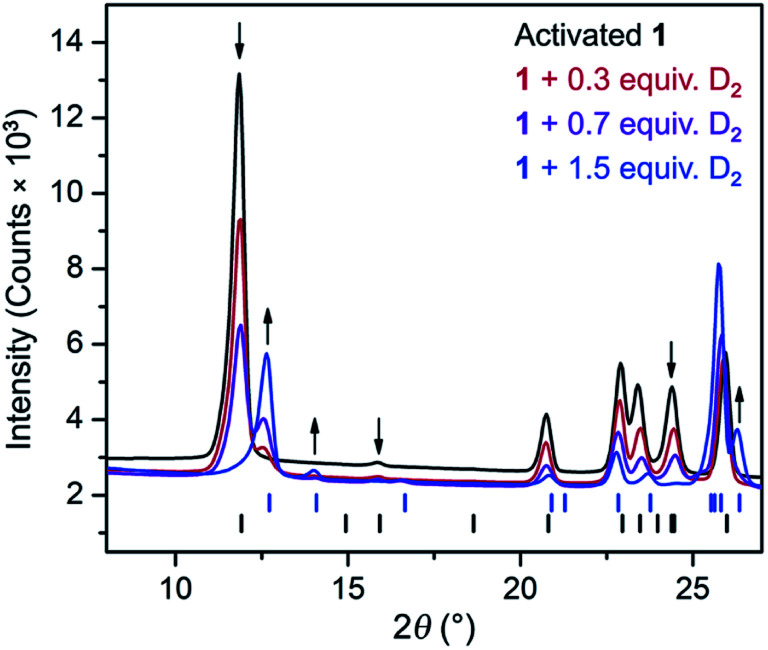
Rietveld refinement fits of powder neutron diffraction patterns (*λ* = 2.0772 Å, *T* = 9 K) collected for activated **1** and activated **1** dosed with 0.3, 0.7, and 1.5 equiv. of D_2_ per pore. Black (lower) and blue (upper) tick marks indicate calculated Bragg peak positions for **1** and the D_2_ adsorbed phase, **1–D_2_**, respectively. Arrows indicate peaks that best illustrate the conversion of **1** to **1–D_2_** with increasing D_2_ loading.

The CH_4_ adsorption isotherm for **1** obtained at 195 K exhibits a steep uptake similar to that characterized for H_2_ at low pressures, again indicative of strong interactions between CH_4_ and the binding pockets of the framework (Fig. 3). A dual site Langmuir model was used to simultaneously fit isotherm data collected at 195, 273, 298, and 308 K, and the Clausius–Clapeyron equation was then employed to calculate a value of *Q*_st_ = –24.8 kJ mol^–1^ at low loadings (see ESI, Fig. S5†). Notably, this value surpasses that determined for Zn_4_O(bdc)_3_ (MOF-5; *Q*_st_ = –12.3 kJ mol^–1^) and even values for frameworks with snugly fitting pore window, such as Cu_2_(btc)_3_ (*Q*_st_ = –17.1 kJ mol^–1^), or strongly polarizing open metal cation sites, as in Ni_2_(dobdc) (dobdc^4–^ = 2,5-dioxido-1,4-benzenedicarboxylate; *Q*_st_ = –20.6 kJ mol^–1^).^41^,^45^ It is clear that the binding pockets in U(bdc)_2_ can strongly interact with both H_2_ and CH_4_, despite their different sizes, which suggests that the framework may distort or flex to optimize interactions with different guest molecules. In order to study the framework–guest interactions in more detail, we turned to *in situ* gas-dosing powder neutron diffraction. The powder neutron diffraction pattern of activated **1** at 9 K was first collected as a reference for gas dosing experiments (see ESI, Fig. S9†). Dosing with 0.3 equiv. of D_2_ per pore results in clear changes in the powder pattern, particularly visible at low values of scattering angle 2*θ* (Fig. 4 and S10†). Specifically, the reflections of **1** decrease in intensity while a second set of peaks arises, ascribed to a new crystalline phase **1–D_2_**. Upon increasing the loading to 0.7 equiv. of D_2_, both phases are still present, although the peaks of **1** diminish further and the peaks of newly formed **1–D_2_** gain in intensity (Fig. 4 and S11†). The two phases coexist up to a loading of at least 1.5 equiv. D_2_ per pore (see ESI, Fig. S12†), and their interconversion is best followed by evaluating the high intensity, low angle peaks at 2*θ* ≈ 11.85° for **1** and at 2*θ* ≈ 12.63° for **1–D_2_** in Fig. 4. These data strongly suggest that cooperative effects drive an adsorbate-induced framework distortion from **1** to **1–D_2_**. Such a mechanism is in contrast to a gradual and homogeneous uptake of D_2_, or a gradually changing degree of distortion depending on the D_2_-loading. As a result, before achieving saturation loading, some individual crystallites of the sample will be distorted, such that both binding pockets per pore are occupied with a D_2_ molecule, while others will remain in the activated structure of **1**. At the highest D_2_ dosing level of 2.5 equiv. per pore, the diffraction pattern of the sample contains only reflections associated with **1–D_2_** (see ESI, Fig. S13†).

*In situ* powder neutron diffraction experiments were also carried out by dosing **1** with 0.7 and 1.5 equiv. of CD_4_ (Fig. S14 and S15†). The data from these experiments suggest a similar cooperative transformation from **1** to an adsorbed phase **1–CD_4_**. Rietveld refinements were applied to all powder neutron diffraction data (see Section 6 in the ESI†) in order to elucidate adsorbate-induced structural deformations and characterize specific adsorption sites for D_2_ and CD_4_. Selected unit cell parameters determined for the different structures are summarized in Table 1. Based on unit cell volume, **1** contracts to a greater extent to accommodate D_2_ than it does in the presence of CH_4_.

**Table 1:** Unit cell parameters (space group *C*2/*c*) for activated **1** and **1** dosed with different adsorbates, along with selected angles describing the pore deformation upon guest adsorption. Angles *φ*_1_ and *φ*_2_ describe the U···U···U angles of the idealized parallelogram spanned by the four corner uranium atoms within the crystallographic *ab-*plane of each pore, as illustrated in Fig. 5a. Angles *ω*_1_ and *ω*_2_ describe the dihedral angle between O–U···U–O planes and O–C–O planes at each end of a bdc^2–^ linker, as illustrated in Fig. 5b for **1** and **1–D_2_**. One standard deviation for all values is given in parentheses

Sample	*a* (Å)	*b* (Å)	*c* (Å)	*V* (Å^3^)	*φ*_1_ (°)	*φ*_2_ (°)	*ω*_1_ (°)	*ω*_2_ (°)
**1**	17.587(1)	12.812(1)	9.2999(5)	1902.1(2)	77.1(1)	102.902(3)	151(1)	168.4(9)
**1–D_2_** (0.3 equiv.)	18.361(7)	11.35(1)	9.335(2)	1782(2)	67.51(1)	112.491(9)	148.7(3)	159.6(3)
**1–D_2_** (0.7 equiv.)	18.402(3)	11.324(5)	9.333(1)	1781.4(8)	67.4(2)	112.576(1)	130(2)	175(3)
**1–D_2_** (1.5 equiv.)	18.456(1)	11.233(1)	9.3508(4)	1775.8(2)	66.86(6)	113.143(1)	141.4(8)	170(1)
**1–D_2_** (2.5 equiv.)	18.665(1)	10.9486(8)	9.3838(5)	1754.5(2)	65.03(6)	114.973(1)	138.5(4)	174.9(5)
**1–CD_4_** (1.5 equiv.)	18.031(1)	11.9665(7)	9.3206(4)	1839.3(2)	71.52(5)	108.483(1)	142(3)	171.1(9)
**1–DMF**	18.2658(7)	12.0252(5)	9.3579(3)	1865.6(1)	71.63(4)	108.373(1)	166(11)	164(4)

**Fig. 5:**
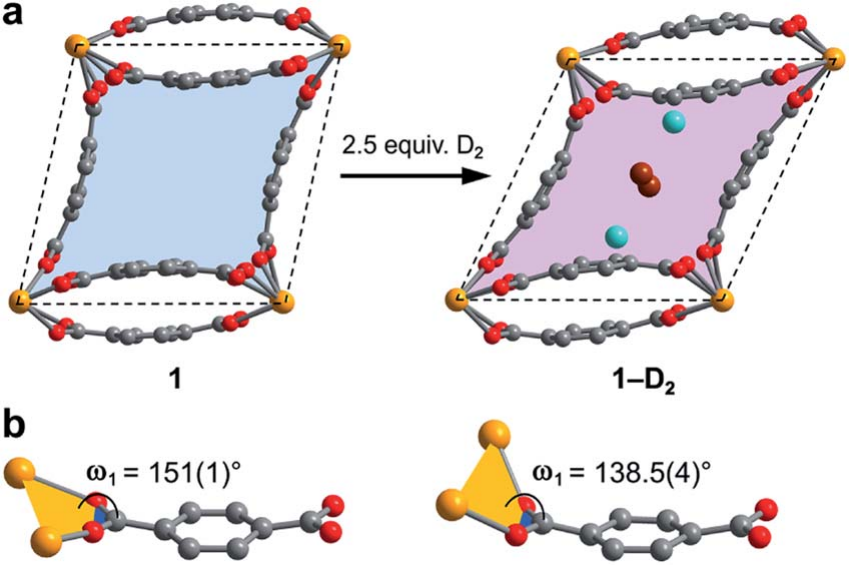
(a) Illustration of pores in **1** and **1–D_2_** (dosed with 2.5 equiv. D_2_ per pore). The pore contraction upon D_2_ dosing is highlighted by the colored blue and purple areas within the crystallographic *ab*-plane. Tilting of the pores to facilitate contraction is illustrated by idealized parallelograms (dashed lines) with corners defined by uranium atoms. Adsorbed D_2_ molecules inside the pores of **1–D_2_** are represented as aquamarine spheres for adsorption site I and as brown spheres for adsorption site II. Each D_2_ super atom at site II is shared between two neighboring pores, accordingly the two depicted site II super atoms together account for an occupancy of one D_2_ molecule per pore. (b) Comparison of the dihedral angles *ω*_1_ as described in the text (*ω*_2_ is not shown, but is the corresponding angle at the other end of the linker), representing the main structural distortion undergone by **1** upon adsorption of D_2_. Orange, red, and grey spheres represent U, O, and C atoms, respectively.

Surprisingly, a structural comparison of **1** with **1–D_2_** and **1–CD_4_** reveals an almost constant coordination environment around the uranium nodes in each phase. The framework flexibility instead relies on a tilting of the parallelepipedal pores, which is clearly seen by comparing the pore geometries of **1** and **1–D_2_** dosed with 2.5 equiv. D_2_, as shown in Fig. 5a. Here, each structure is overlaid with an idealized parallelogram with corners defined by the uranium ions. Dosing with 2.5 equiv. of D_2_ results in a change of the idealized parallelogram angles, *φ*_1_ and *φ*_2_, from 77.1(1)° and 102.902(3)° in **1** to 65.03(6)° and 114.973(1)° in **1–D_2_**. In order to accommodate this rearrangement, the two unique dihedral angles, *ω*_1_ and *ω*_2_, between the bdc^2–^ O–C–O planes and the neighboring O–U···U–O planes adjust from 151(1)° and 168.4(9)° in **1** to 138.5(4)° and 174.9(5)° in **1–D_2_** (Fig. 5b).

The resulting hinge-type bending between the UO_8_ nodes and linkers is analogous to the change that occurs in the structure of the flexible framework Cr(OH)(bdc) upon water adsorption.^53^ In particular, water adsorption is accompanied by a unit cell volume decrease from 1486.1 to 1012.6 Å^3^, as well as a decrease of the symmetrical dihedral angles, *ω*, from 179.8° to 162.3°. The analogous idealized Cr···Cr···Cr angles *φ*_1_ and *φ*_2_ in Cr(OH)(bdc) change drastically from 75.9° and 104.2° (activated) to 44.8° and 135.2° (hydrated).^56^ Interestingly, in distinct contrast to **1**, Cr(OH)(bdc) distorts very little upon interaction with D_2_ (<4 equiv. per pore), adopting a symmetric dihedral angle *ω* = 178.4° and Cr···Cr···Cr angles of 80.6° and 99.4°, concomitant with a very small unit cell volume increase to 1534.5 Å^3^.^58^,^71^ The change in the structure of **1** upon dosing with 2.5 equiv. of D_2_ results in a much more drastic change in unit cell volume, from 1902.1(2) to 1754.5(2) Å^3^. We rationalize that the greater deformation of **1** arises as a result of its better ability to enshroud H_2_ within its pores, which leads to a greater adsorption enthalpy (*Q*_st_ = –8.6 kJ mol^–1^*vs.* –6.9 kJ mol^–1^ for Cr(OH)(bdc)),^72^ and a larger driving force for structural rearrangement. Thus, the smaller pores within the framework of **1** are able to optimize binding through multiple stabilizing interactions, whereas the comparably large pores of Cr(OH)(bdc) provide fewer contacts.

**Fig. 6:**
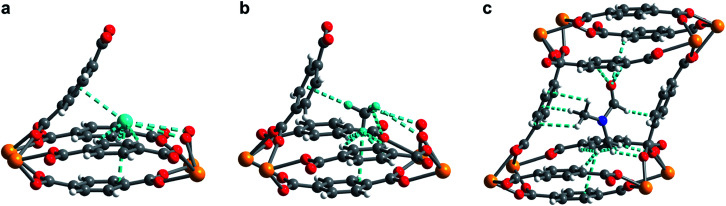
(a) Neutron powder diffraction structure of D_2_ adsorbed at site I in **1–D_2_** after dosing **1** with 2.5 equiv. D_2_ per pore. (b) Neutron powder diffraction structure of CD_4_ adsorbed at site I in **1–CD_4_** after dosing **1** with 1.5 equiv. CD_4_ per pore. (c) Powder X-ray diffraction structure of DMF adsorbed inside the pore of U(bdc)_2_. For clarity, only selected interactions of DMF with the framework are depicted. All framework–guest interactions are depicted as aquamarine colored dashed lines. For clarity in (a) and (b), the pore is truncated and cut in half diagonally, showing only one of the two adsorption pockets of the pore. Orange, red, grey, blue, aquamarine, and white spheres represent U, O, C, N, D, and H atoms, respectively.

In order to elucidate the hydrogen binding sites in **1**, we treated the D_2_ molecules as “super atoms” in our analysis of the diffraction data (see ESI, Section 6†).^73^,^74^ We first analyzed the structure of **1** loaded with 1.5 equiv. of D_2_ per pore (corresponding to less than one D_2_ per pocket) to enable an accurate structure determination in the absence of adsorbate–adsorbate interactions.^75^–^77^ As expected, the D_2_ super atoms were located in both binding pockets of each pore, with an occupancy of 75% per site. Each D_2_ super atom is situated within van der Waals contact distance of three H atoms of bdc^2–^ linkers, the π-system of the outer pocket-capping bdc^2–^ linker, and two oxygen atoms of the UO_8_ coordination polyhedron (Fig. 6a). The D_2_···H contact distances of 2.96(1), 2.98(1), and 3.15(2) Å indicate moderately strong van der Waals interactions.^74^,^78^,^79^ The distance from D_2_ to the centroid of the nearest benzene ring is 3.45(1) Å, which is indicative of a modest D_2_···π interaction,^80^ while the closest D_2_···O contact is 3.59(1) Å. In the structure of **1** dosed with 2.5 equiv. of D_2_, an additional D_2_ molecule was located in the center of the pore (site II, see Fig. 5a). The D_2_ molecules at site II are stabilized by four symmetry equivalent D_2_···D_2_ interactions at a distance of 3.10(1) Å, as well as by weak C–H···D_2_ contacts with the linkers (3.31(3) and 3.55(1) Å). As discussed above, in order to accommodate these interactions, the pores of **1** contract significantly around the D_2_ molecules, decreasing the unit cell volume by as much as 7.8% and shrinking the binding pocket diameter from 5.0 to 3.6 Å (see ESI, Section 6†). The crucial role of this structural distortion is further exemplified by considering a hypothetical D_2_ super atom at the fractional coordinates of site I in fully activated **1**. In this environment, the D_2_···framework interactions are elongated beyond meaningful van der Waals contact distances (see ESI, Section 6†).

Rietveld refinement of *in situ* powder neutron diffraction data collected for **1** dosed with 1.5 equiv. of CD_4_ confirmed that the molecule occupies the same adsorption pocket as D_2_ (site I). Due to the larger size of CD_4_ relative to D_2_, a less pronounced contraction of the framework is sufficient to enable similar host–guest contacts. The unit cell volume of **1–CD_4_** contracts by 3.4% to 1839.3(2) Å^3^, resulting in a binding pocket diameter of 4.1 Å. Notably, the adsorbed CD_4_ molecules are well-ordered as a result of a large number of specific host–guest interactions (Fig. 6b). The nearest D···arene distances are 2.740(9) Å (side wall of the pore) and 3.407(8) Å (outer linker of bowl-shaped cap). These relatively short distances are consistent with those determined previously from studies of methane adsorption on benzene (2.1–3.8 Å) and support the characterized orientation of CD_4_ inside the pore of U(bdc)_2_.^45^,^81^–^83^ Additional D···H van der Waals contacts at 2.57(1), 2.69(2), and 2.81(2) Å stabilize and orient the adsorbed CD_4_ molecules within the binding pocket. Adsorbed CD_4_ further interacts with three O atoms of two independent UO_8_ nodes at distances of 3.10(2), 3.25(1), and 3.50(1) Å. Lastly, adsorbate–adsorbate interactions based on D···D contacts at 2.84(1), 3.10(1), and 3.20(1) Å stabilize CD_4_ inside each pore.^84^,^85^ Together, the wealth of stabilizing contacts explains the competitively high heat of adsorption (*Q*_st_ = –24.8 kJ mol^–1^) for methane in U(bdc)_2_. It is worth noting that methane is also expected to occupy a second adsorption site at higher loadings, as seen for D_2_. This observation is supported by the CH_4_ adsorption data shown in Fig. 3. Here, initial steep uptake is associated with saturation of the binding pockets of site I until a loading of 3.5 mmol g^–1^. The onset of far more gradual CH_4_ uptake to 4.1 mmol g^–1^ at 1.2 bar suggests additional methane adsorption at a second, weaker binding site.

Finally, we sought to study the distortion of **1** in the presence of an even larger guest molecule and prepared crystals of **1–DMF** by soaking the framework in dry DMF (kinetic diameter of 5.5 Å).^86^ The structure of **1–DMF** was determined from Rietveld refinement of powder X-ray diffraction data obtained at 298 K (see ESI, Fig. S8†). The framework indeed contracts to optimize interactions with DMF, but the unit cell volume decreases by only 1.9% (compared to 7.8% and 3.4% in the cases of 2.5 equiv. D_2_ and 1.5 equiv. CD_4_, respectively) and the pocket diameter only decreases to 4.2 Å. While two molecules of the smaller guests D_2_ and CD_4_ can simultaneously occupy the two binding pockets in each pore, the larger DMF molecule occupies the whole pore space, bridging both pockets (Fig. 6c). As a result, DMF is stabilized by seven of the eight bdc^2–^ linkers that form the surrounding pore. While precise contact distances are obscured by disorder of DMF over two positions in the structure, the general identity of host–guest interactions in **1–DMF** is clear. In particular, DMF binds through eight H···H contacts, three C–H···π-interactions, three O···H contacts involving the carbonyl and arene C–H moieties, and interactions of two DMF C–H groups with O atoms of two UO_8_ nodes (all distances between 2 and 4 Å).

## Conclusions

Porous adsorbents with small binding pockets can engage in strong, selective host–guest interactions, and are therefore of interest for applications including gas capture and storage. However, it remains a significant challenge to tune a binding pocket structure to optimize interactions with a specific target molecule. In this work, we show that combining moderate flexibility with small pores in the new flexible metal–organic framework U(bdc)_2_ (**1**) eliminates the need for precise tuning of pore geometry. Indeed, this material is capable of uniquely adjusting its pore and binding pocket geometry to optimize host–guest interactions in the presence of even very weakly adsorbing molecules, such as H_2_ and CH_4_. Temperature-dependent H_2_ and CH_4_ adsorption isotherms yielded isosteric heats of adsorption of –8.6 and –24.8 kJ mol^–1^ for H_2_ and CH_4_, respectively, confirming comparatively strong interactions of **1** with both gases, despite their different sizes. *In situ* powder neutron diffraction experiments with D_2_ and CD_4_ revealed that cooperative effects drive a spontaneous adjustment of the binding pockets in **1** to generate multiple stabilizing interactions between each adsorbate and the framework, which are not achieved without pore contraction. Altogether, our results demonstrate the utility of frameworks featuring self-adjusting binding pockets that can flex in response to different guest molecules. The design principles applied for our model material **1** are transferable to non-radioactive frameworks and suggest further exploration of such materials will be advantageous in the search for adsorbents with improved gas storage properties.

## Conflicts of interest

There are no conflicts to declare.

## Supplementary Material

Supplementary information

Supplementary information

Crystal structure data
