# Supplementary material for: Self-adjusting binding pockets enhance H2 and CH4 adsorption in a uranium-based metal–organic framework
Source: Chem Sci. 2020 May 27;11(26):6709–16. doi: 10.1039/d0sc02394a (PMC7473405; doi:10.1039/d0sc02394a)
Supplement: Supplementary file 2 [file SC-011-D0SC02394A-s002.pdf]

Electronic Supplementary Material (ESI) for Chemical Science.
This journal is © The Royal Society of Chemistry 2020

Supplementary Information for:

Self-adjusting binding pockets enhance H_2 and CH_4 adsorption in a uranium-based metal–organic framework

Dominik P. Halter, Ryan A. Klein, Michael A. Boreen, Benjamin A. Trump, Craig M. Brown, and Jeffrey R. Long*

*correspondence to: jrlong@berkeley.edu

Table of Contents

1. General experimental procedures 2
2. Synthesis of U(bdc)$_2$ (**1**) 3
3. Surface area determination and gas adsorption experiments 4
4. Thermogravimetric analysis 7
5. Powder X-ray diffraction 8
6. Powder neutron diffraction 9
7. Single crystal X-ray diffraction 14
8. References 17

1. General experimental procedures

General procedures. The synthesis of U(bdc)$_2$ (**1**) was performed under inert gas atmosphere using standard Schlenk line techniques and a glove box equipped with argon. Terephthalic acid was purchased from commercial vendors[†] and dried at 80 °C under reduced pressure (approx. 5×10^{-2} mbar) prior to use. *N*,*N*-Dimethylformamide (DMF) was purchased from Fischer Scientific (purity > 99.8 %, water content < 0.15 %) and degassed by sparging with argon prior to use. Anhydrous DMF was obtained by drying the degassed DMF over 4 Å activated molecular sieves. Anhydrous methanol was purchased from Sigma Millipore and used as received. UI_4(1,4-dioxane)$_2$ was synthesized according to the literature procedure.[1] Ultra-high purity grade (99.999% purity) helium, nitrogen, hydrogen, and methane were used for all adsorption measurements.

Gas adsorption measurements. Gas adsorption data were obtained using instrumentation and protocols that closely follow a previously used approach,[2] and are reproduced here for completeness with new details relevant to this work.
Gas adsorption isotherms for pressures in the range of 0 to 1.2 bar were measured using a Micromeritics ASAP 2020 or 2420 instrument. Activated samples were transferred under a N_2 atmosphere to pre-weighed analysis tubes, which were capped with a TranSeal. Each sample was evacuated on the ASAP instrument until the outgas rate was less than 3 μbar min^{-1}. The evacuated analysis tube containing degassed sample was then carefully transferred to an electronic balance and weighed to determine the mass of sample (typically 100–150 mg). The tube was then fitted with an isothermal jacket and transferred back to the analysis port of the ASAP instrument. The outgas rate was again confirmed to be less than 3 μbar min^{-1}. Langmuir surface areas were determined by measuring N_2 adsorption isotherms in a 77 K liquid N_2 bath and calculated using the Micromeritics software, assuming a value of 16.2 Å^2 for the molecular cross-sectional area of N_2.

Elemental analysis was measured at the Microanalytical Facility at the College of Chemistry, University of California, Berkeley.

Thermogravimetric analysis (TGA) was performed with a TA Instruments TGA Q50 Thermogravimetric Analyzer at a ramp rate of 2 °C per minute under nitrogen flow.

Laboratory powder X-ray diffraction. Patterns were collected using a Bruker AXS D8 Advance diffractometer using Cu K_α radiation (λ = 1.5418 Å).

Synchrotron powder X-ray diffraction. Synchrotron powder X-ray diffraction data for **1** and **1–DMF** were collected on beamline 17-BMB at the Advanced Photon Source (APS) at Argonne National Laboratory (λ = 0.45246 Å). Samples were sealed in a quartz capillary, which was then sealed inside a Kapton tube with epoxy resin. Data was collected using an amorphous Si area detector with the two-dimensional patterns reduced to one-dimensional data with GSAS-II.[3] Measurements were conducted at room temperature ($T \approx 298$ K), with samples hermetically sealed throughout the experiment.

Powder neutron diffraction. Powder neutron diffraction experiments were performed at the National Institute of Standards and Technology Center for Neutron Research (NCNR) using the high-resolution neutron powder diffractometer, BT1. Data were collected using a Ge(311) monochromator with an in-pile 60' collimator, corresponding to a neutron wavelength of λ = 2.0772 Å. Specific experimental details are reported together with the data in the powder neutron diffraction section (Section 6).

Single crystal X-ray diffraction. Diffraction data were obtained at the Advanced Light Source (ALS), Lawrence Berkeley National Lab, Berkeley, CA, station 12.2.1, using a silicon monochromated synchrotron radiation beam of 17 keV (λ = 0.7288 Å). Specific experimental details are reported together with the data in the single crystal X-ray diffraction section (Section 7).

[†] Certain commercial equipment, instruments, or materials are identified in this document. Such identification does not imply recommendation or endorsement by the National Institute of Standards and Technology, nor does it imply that the products identified are necessarily the best available for the purpose.

2. Synthesis of $U(bdc)_2$ (1)

A glass vial was placed into an autoclave reactor (*Parr Instrument Company*, model 4749) and charged with UI_4(1,4-dioxane)$_2$ (242 mg, 0.263 mmol), terephthalic acid (130 mg, 0.79 mmol), and degassed, non-dried DMF (4.0 mL). The reaction mixture was briefly stirred with a spatula to dissolve all solids, before the vessel was sealed under argon and heated in an oven to 140 °C for 72 h. The crude product formed as emerald green needles, and a portion of the crystals were picked directly from the mother liquor for single-crystal X-ray diffraction analysis. The remaining crystals were transferred into a Schlenk flask, where the mother liquor was removed. Another portion of crystals were crushed and analyzed as a bulk sample using powder X-ray diffraction (see Figure S7). The remaining green solid was soaked four times in dry, degassed DMF (5 mL) at room temperature for 10 h each. A portion of this solid was used for powder X-ray diffraction characterization of the DMF-loaded framework, as described in the main text. The then remaining DMF-soaked green, crystalline solid was isolated by decanting the solvent and then soaked four times in dry methanol (5 mL) at room temperature for 10 h each. After decanting the solvent, the product was dried *in vacuo* at 120 °C for 5 h. Finally, $U(bdc)_2$ was activated by heating it to 260 °C under reduced pressure (2×10^{-6} bar) for 12 h, affording **1** as a green, microcrystalline powder in 79% yield (118 mg, 0.208 mmol).

Elemental Analysis for $U(bdc)_2$ ($C_{16}H_8O_8U$): Calculated: C, 33.94; H, 1.42; N, 0.00. Found: C, 33.56; H, 1.38; N, 0.00.

Note: In the absence of water, we found the reaction of UI_4(1,4-dioxane)$_2$ and H_2(bdc) yields the reported two-dimensional phase $U_2(bdc)_2(DMF)_4$.[4] This material was previously obtained from UCl_4 under otherwise analogous conditions, indicating that H_2O may have a structural directing effect during the synthesis of **1**.

3. Surface area determination and gas adsorption experiments

In preparation for all gas adsorption measurements, U(bdc)$_2$ samples were activated by heating the material to 260 °C under dynamic vacuum until the outgas rate was less than 3 µbar min^{-1} to rigorously desorb all guest molecules from the framework. Additionally, the quality of individual samples was confirmed by N_2 adsorption isotherms at 77 K, which consistently yielded a Langmuir surface area of 497 ± 6 m²/g.

Gas adsorption data were fitted and isosteric heats of adsorption determined following protocols that closely follow a previously used approach[5] and are reproduced here for completeness with new details relevant to this work.

Adsorption isotherm fitting. Adsorption isotherms were fitted with a dual-site Langmuir–Freundlich equation (eq.1), where n is the total amount adsorbed in mmol/g, P is the pressure in bar, $n_{sat,1}$ is the saturation capacity in mmol/g, b_i is the Langmuir parameter in bar^{-1} defined in eq. 2, and v is the Freundlich parameter for the two sites 1 and 2. Experimental adsorption isotherm data for **1** and the corresponding fits show a small discrepancy at higher gas loadings. These may originate from the flexibility of **1** that allows additional small framework deformations as binding site II is occupied at higher gas loadings.

$$n = \frac{n_{sat,1}\, b_1\, P^{v_1}}{1+b_1\, P^{v_1}} + \frac{n_{sat,2}\, b_2\, P^{v_2}}{1+b_2\, P^{v_2}} \quad (1)$$

$$b_i = e^{-S_i}\, e^{\frac{E_i}{RT}} \quad (2)$$

For eq. 2, S_i is the entropy of adsorption at saturation in units of R and E_i is the enthalpy of adsorption in kJ/mol, for site *i*. Also for eq. 2, R is the ideal gas constant and T is the temperature. The fitted parameters for all gases for can be found in tables S1 and S2.

Isosteric heat of adsorption calculations. Using dual-site Langmuir-Freundlich fits, the isosteric heat of adsorption, $-Q_{st}$, was calculated for each sample as a function of the amount of gas adsorbed using the Clausius-Clapeyron relation (eq. 3), where R is the ideal gas constant, P is the pressure, and T is the temperature.

$$-Q_{st} = RT^2 \left(\frac{\partial \ln P}{\partial T}\right)_n \quad (3)$$

For multi-site Langmuir-Freundlich models, it is necessary to calculate the loading dependence of the isosteric heat of adsorption. As written, multi-site Langmuir-Freundlich equations specify the amount adsorbed as a function of pressure, while the pressure as a function of the amount adsorbed is needed to use the Clausius-Clapeyron relation. To calculate the isosteric heat of adsorption for evenly spaced loadings, each multi-site Langmuir equation was solved for the pressures that correspond to specific loadings of a given gas in the range 77 to 87 K for H_2, and 195 to 308 K for CH_4. These calculated pressures were then used with eq. 3 to determine the heat of adsorption as a function of the total amount of gas adsorbed.

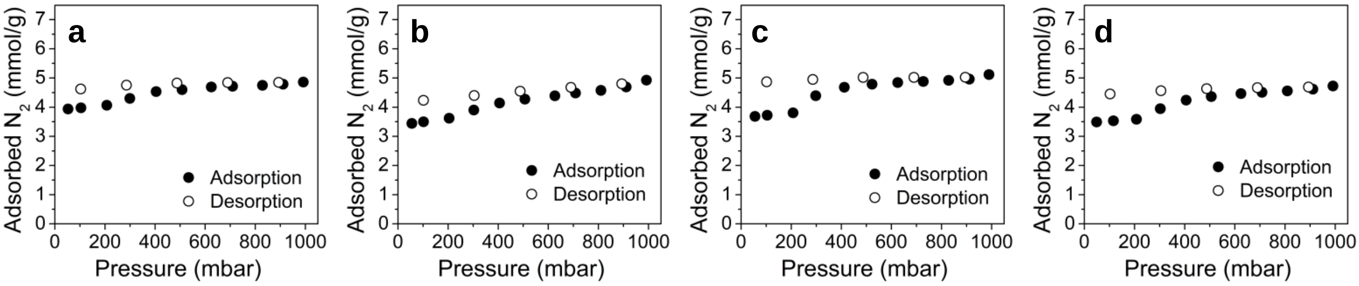

Figure S1. (a – d) 77 K N_2 adsorption isotherms of four independent samples of activated **1**. The Langmuir surface areas were calculated as described in section 1, and determined at A: 494 m^2/g, B: 489 m^2/g, C: 514 m^2/g, D: 490 m^2/g.

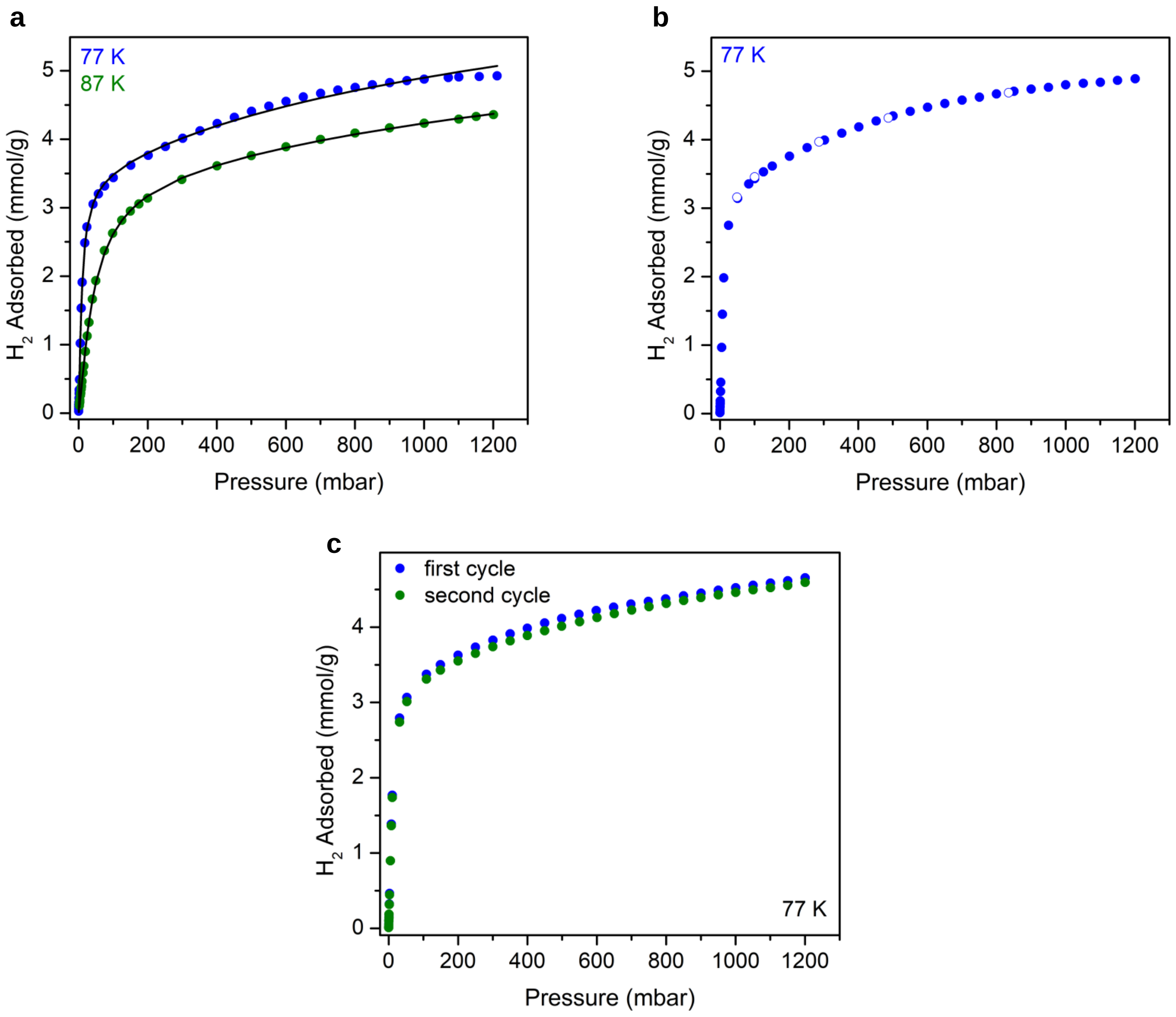

Figure S2. (a) Hydrogen adsorption data obtained for **1** at 77 and 87 K (blue and green symbols, respectively) along with fits to a dual-site Langmuir-Freundlich model (black lines). (b) Isotherms of H_2 adsorption (solid circles) and desorption (open circles) in **1** at 77 K. (c) Hydrogen adsorption data obtained for two consecutive adsorption experiments measured with the same sample of **1** at 77 K. In between the measurements, the sample was reactivated following the reported procedure. The nearly identical H_2 uptake and isotherm shapes strongly suggest that H_2 adsorption in **1** is fully reversible and that the material is stable to the activation conditions.

Table S1. Dual-site Langmuir-Freundlich fit parameters for H_2 adsorption in **1.**

	**77 K**	**87 K**
$\boldsymbol{q}_{\mathbf{sat,1}}$	3.25	3.18
$\boldsymbol{b}_{\mathbf{1}}$	481	60.0
$\boldsymbol{\nu}_{\mathbf{1}}$	1.30	1.29
$\boldsymbol{q}_{\mathbf{sat,2}}$	4.50	4.50
$\boldsymbol{b}_{\mathbf{2}}$	0.581	0.327
$\boldsymbol{\nu}_{\mathbf{2}}$	0.826	0.813

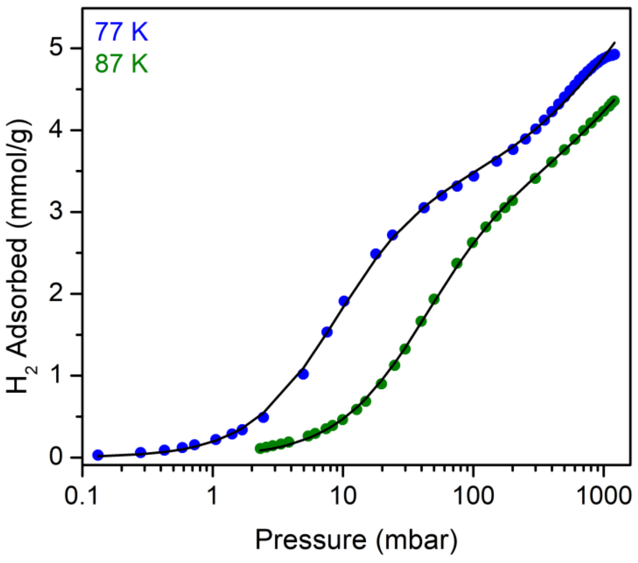

Figure S3. Hydrogen adsorption data obtained for **1** at 77 and 87 K (blue and green symbols, respectively) along with fits to a dual-site Langmuir-Freundlich model (black lines), plotted on a logarithmic scale.

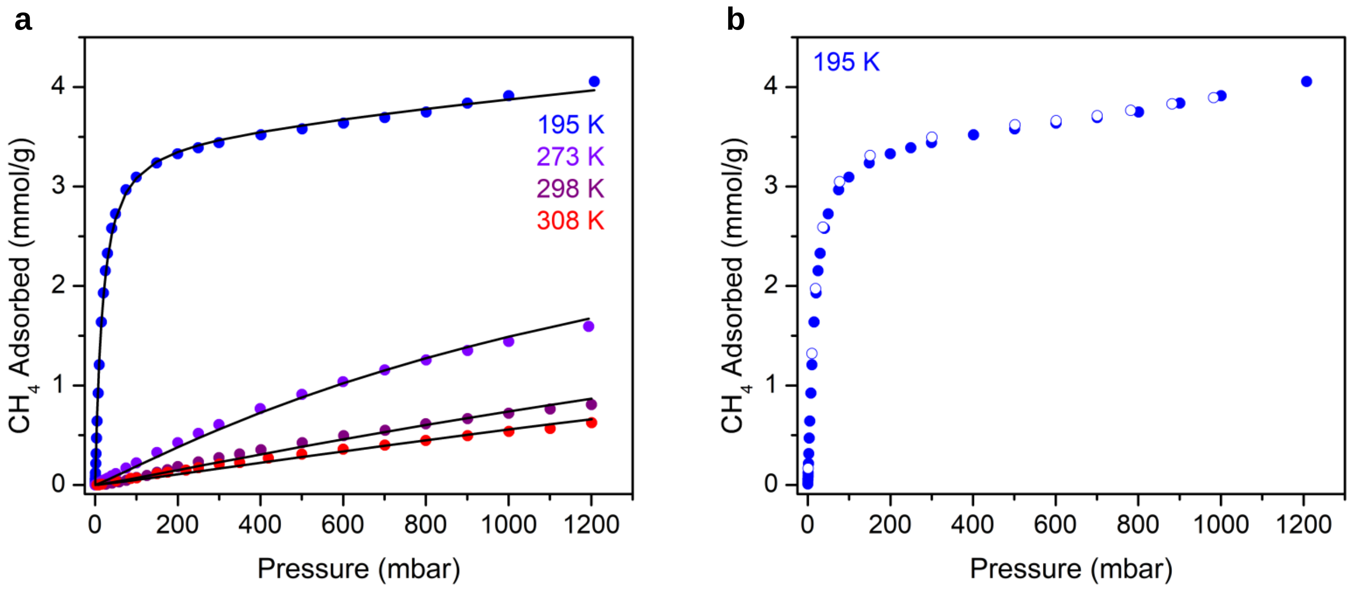

Figure S4. CH_4 adsorption data obtained for **1** at 195, 273, 298, and 308 K (colored symbols) along with fits to a dual-site Langmuir-Freundlich model (black lines). (b) Isotherms of CH_4 adsorption (solid circles) and desorption (open circles) in **1** at 195 K.

Table S2. Dual-site Langmuir-Freundlich fit parameters for CH_4 adsorption in **1**.

	**195 K**	**273 K**	**298 K**	**308 K**
$q_{sat,1}$	3.52	3.52	3.52	3.52
b_1	81.9	0.680	0.249	0.174
ν_1	1.10	1.10	1.10	1.10
$q_{sat,2}$	2.00	2.00	2.00	2.00
b_2	0.249	0.0302	0.0194	0.0166
ν_2	1.30	1.30	1.30	1.30

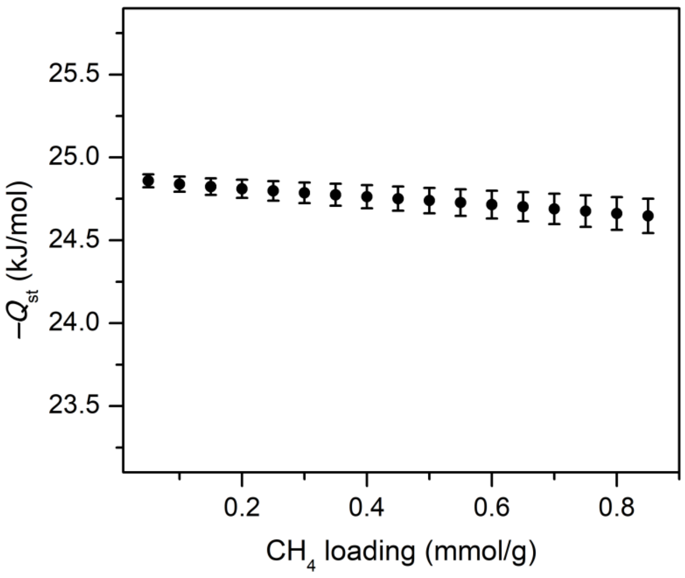

Figure S5. Isosteric heat of adsorption of CH_4 in **1**, plotted as a function of gas loading (black circles) together with standard errors (black bars).

4. Thermogravimetric analysis

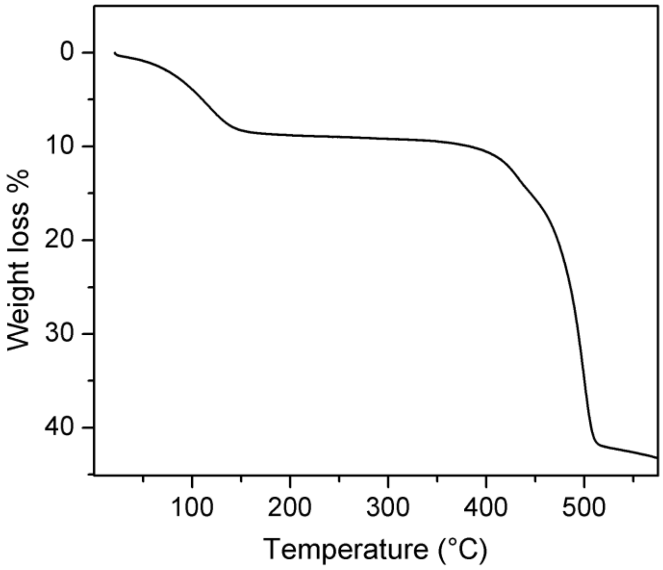

Figure S6. Thermogravimetric analysis plot for **1–H_2O** showing a step for the loss of pore water (9.0 wt%, corresponding to 3.2 H_2O per pore; inflection point at 120 °C) and a decomposition step (inflection point at 500 °C).

5. Powder X-ray diffraction

The Rietveld refinement of the synchrotron X-ray powder diffraction pattern of **1–DMF** used rigid body models for the bdc^{2-} ligands and the DMF molecules. The position of the DMF molecules in the framework was found using simulated annealing in real space using Topas Academic,[6] followed by a systematic Rietveld refinement of all atomic positions and the occupancy of the solvent molecules.[7]

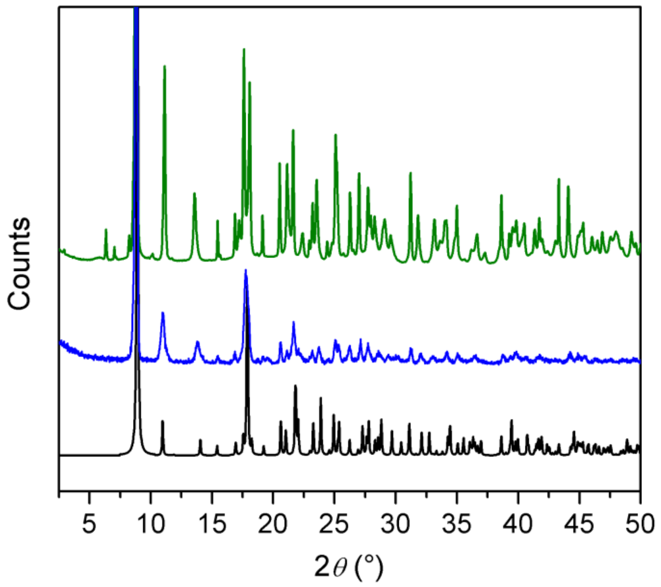

Figure S7. Powder X-ray diffraction patterns of **1–H$_2$O** as generated from single crystal data (black), of **1–H$_2$O** as synthesized (blue), and fully activated **1** (green). Experimental powder patterns were recorded at 298 K and are reported for λ = 1.5418 Å. It should be noted that the three additional low-intensity peaks at 2 Θ = 6.3°, 7.0° and 8.2° in the diffraction pattern of activated **1** (green) are only resolved with high intensity synchrotron radiation. They likely originate from a non-trivial unit cell expansion due to disorder in the flexible framework, defect sites, or possibly due to minimal damage of the framework during the activation procedure.

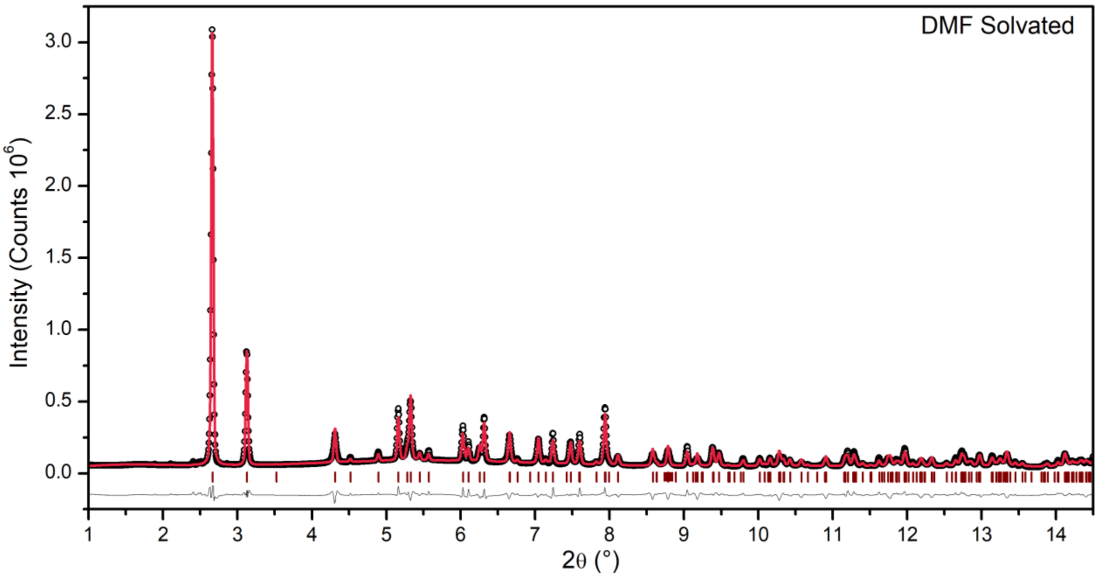

Figure S8. Rietveld refinement of the powder X-ray diffraction pattern obtained for **1–DMF** (λ = 0.45246 Å). Black circles show the experimental diffraction pattern and the red line represent the calculated diffraction pattern, respectively. The gray line represents the difference between observed and calculated patterns, and the brown tick marks indicate calculated Bragg peak positions. T = 298 K; λ = 0.45246 Å; Figures-of-merit: R_{wp} = 7.57, R_p = 5.54, R_{exp} = 1.02, GoF = 7.43.

6. Powder neutron diffraction

Data collection. 0.415 g of pristine crystalline powder of activated **1** was loaded into a cylindrical aluminum sachet and then placed into a cylindrical vanadium can (inner diameter = 6.0 mm). The sample can was sealed with an indium O-ring and a lid with a capillary gas line and a packless valve in a helium-filled glovebox equipped with oxygen and water sensors. The sample was then evacuated at room temperature to approximately 10^{-6} mbar using a turbomolecular pump, mounted in a bottom-loading closed-cycle refrigerator and cooled under static vacuum. Data were collected for the activated material under static vacuum at 9 K over the course of approximately 24 h. The sample was then warmed to 295 K (subsequent gas dosing was performed at 80 K) and dosed with D_2 gas from a known volume (corresponding to ratios of 0.3, 0.7, 1.5, and 2.5 D_2 molecules per pore of the MOF), cooled to base temperature of 9 K and measured for approximately 20 h, each. Upon cooling, the outboard pressure indicated the D_2 gas had been adsorbed well above base temperature except for the 2.5 equivalents D_2 loading, where the adsorption capacity appeared to be reached close to 20 K. After the D_2 dosing experiments, the sample was re-activated at 295 K under dynamic vacuum, dosed with 0.7 equiv. CD_4, and then cooled to 7 K to collect for approximately 20 h. The sample was then warmed to 200 K to further dose with CD_4 until a total loading of 1.5 equiv. CD_4 per MOF pore was reached. The sample was then cooled back to 7 K and another powder pattern was measured at this dosing level.

Structure solutions. The powder diffraction data were analyzed using Topas Academic[6] and EXPGUI/GSAS.[3,8] Initial Pawley fits[9] indicated that the activated structure was in a *C*2/*c* space group. Upon gas dosing, extra peaks emerged in the powder patterns that could be ascribed to a new crystalline phase with a slight distortion of the parent structure but still within the *C*2/*c* space group. The lattice parameters of the gas-dosed structures differ based on the identity of the adsorbate but change very little as a function of concentration of that adsorbate (Table S3).

Rietveld refinements[10] of the powder data (Figures S8–S15) yielded the structures of the respective samples. These refinements used bond length and angle restraints for the bdc^{2-} ligands and CD_4 gas, as well as a super-atom approach for D_2 molecules.[11–13] The super-atom approach treats the D_2 molecules as point scatterers with double occupancy and large thermal parameters. Fourier difference maps for the gas dosed diffraction data were created using the structure solution for the activated material. The Fourier difference maps revealed the approximate position — and, for the CD_4 molecules, the orientation — of the gas molecules in the framework in real space. Gas molecules with fixed occupancy based on the known dosing concentrations were added to the framework at these positions. Then, the isotropic thermal parameters and positions of the ligand and gas atoms were refined, as well as the occupancy of the gas atoms.

Upon dosing the activated framework **1** with D_2 loadings of less than one molecule per binding pocket (i.e. < 2.0 equiv. per pore), the diffraction data shows the convoluted neutron diffraction patterns of phases **1** and **1–D$_2$**. The molar ratios of both phases change with the gas loading, causing also the scaling factors of both phases to change. At a D_2 loading of 0.3 equiv. the minority phase is **1–D$_2$** and the majority phase is **1**, whereas at a gas loading of 1.5 equiv. D_2, **1** has become the minority phase, as most of it is has been converted to **1–D$_2$** (now the majority phase). Due to their low signal intensity, the minority phases (*i.e.* **1–D$_2$** in the 0.3 equiv. D_2 data, and **1** in the 1.5 equiv. D_2 dosed data) required additional constraints during the structural refinement, whereas the majority phases were refined as described above. At a loading of 0.7 equiv. D_2 both phases **1** and **1–D$_2$** were well enough resolved to be refined using the standard approach described above.

For the weakly diffracting minority phases in the 0.3 equiv. D_2 and 1.5 equiv. D_2 gas-dosed patterns, the thermal parameters and atom positions were not refined. When necessary, the occupancies of the gas molecules were fixed to match the dosed concentration value. The Rietveld refinements also yielded the relative phase fractions of the activated and gas-adsorbed structures. The phase fraction of the activated structure decreased monotonically, while the phase fraction of the gas-adsorbed structure increased monotonically as a function of the gas loading in both the D_2 and CD_4 gas-dosed data sets. At similar gas loadings, the percent conversion from the bare to the gas-dosed structure is similar between the two gases, indicative of a comparable mechanism of structural distortion.

In order to further emphasize how structural distortions support the gas adsorption in **1**, a hypothetical dummy D_2 super-atom was placed in the undistorted activated framework at the fractional coordinates of site I. In this hypothetical undistorted pocket, the distance of D_2 to the centroid of the nearest aromatic ring increased from 3.45(1) Å in **1–D$_2$**, dosed with 1.5 equiv. D_2, to 3.91 Å, basically eliminating all stabilizing interactions. Analogously, the distance from D_2 to the centroid of the nearest triangular face of the UO_8 node elongated from 3.52(2) to 4.01 Å, preventing effective host-guest interactions. We note that although great care was taken in this approach, the required use of fractional coordinates in combination with the reported structural differences between **1** and **1–D$_2$**, introduces unknown but non-negligible uncertainties to the reported contact distances between the generated dummy atom and the experimentally confirmed activated framework structure. Accordingly, these values can only serve as an estimate, although they confirm that the strong adsorption site I – as determined in **1–D$_2$** – would be too distant from all relevant adsorption features of the binding pocket in **1** to allow reasonable interactions without pore contraction.

In order to further describe the binding pocket contraction of **1** in response to different adsorbates, diameters of approximated spherical binding pockets were determined based on crystallographic structure data. First, the geometric centroid of a binding pocket was calculated in each structure. Then, a sphere was generated around this centroid such that the sphere did not make contact with any atom of the pore, based on van der Waals radii. The diameter of the resulting sphere around the centroid was then reported as the approximated binding pocket diameter. Binding pocket diameters were determined at 5.0, 3.6, 4.1, and 4.2 Å for activated **1**, **1–D$_2$** (dosed with 2.5 equiv. D_2), **1–CD$_4$** (dosed with 1.5 equiv. CD_4), and **1–DMF**, respectively.

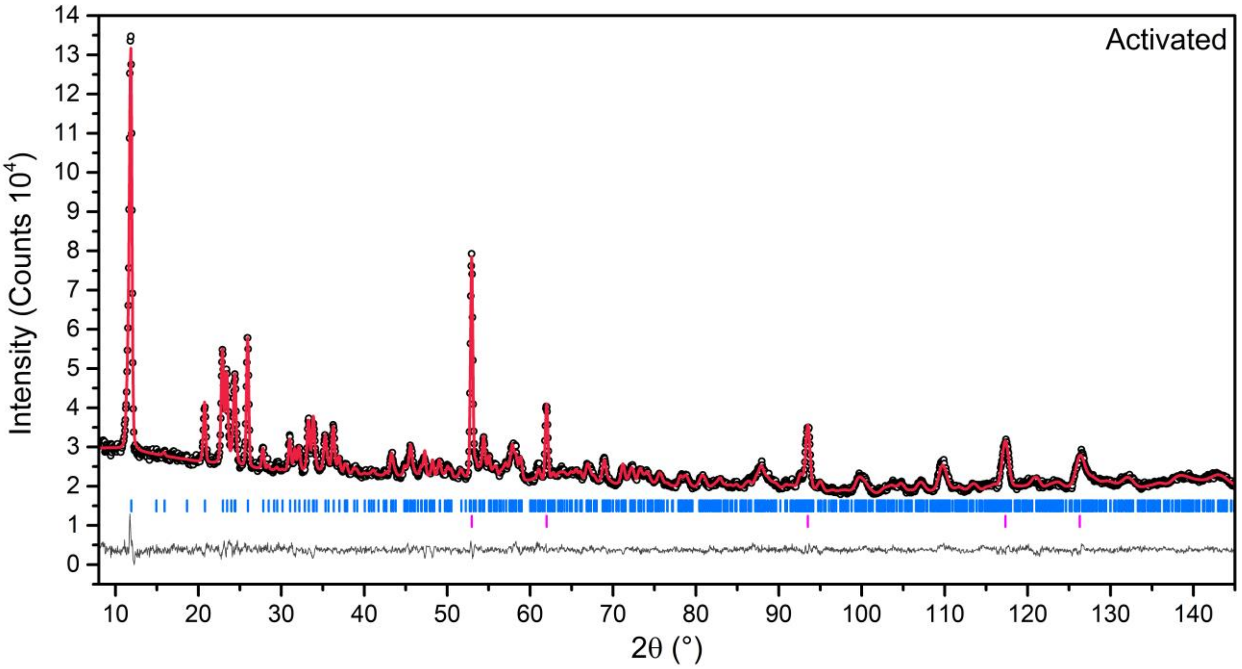

Figure S9. Rietveld refinement of the powder neutron diffraction pattern obtained for activated phase **1** (λ = 2.0772 Å; T = 9 K). Black circles show the experimental diffraction pattern and the red line represents the calculated diffraction pattern. The gray line represents the difference between observed and calculated patterns, the blue tick marks indicate calculated Bragg peak positions of the activated phase **1**, and the pink tick marks indicate reflections from the aluminum sachet.

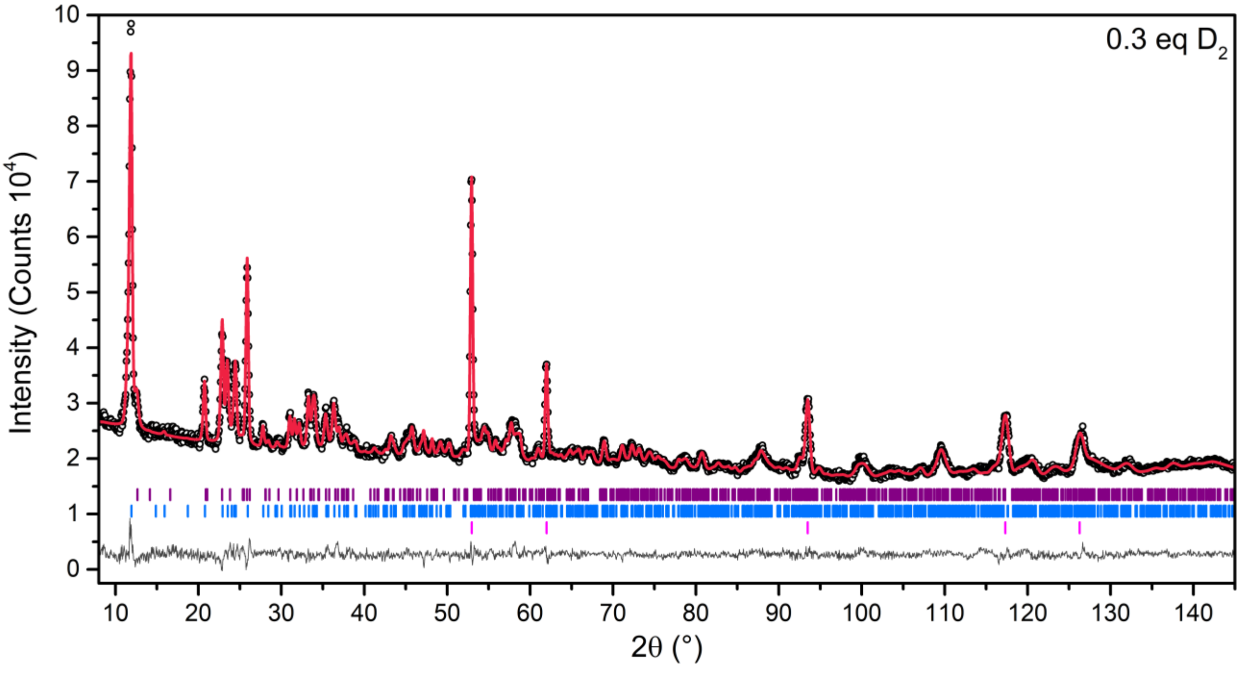

Figure S10. Rietveld refinement of the powder neutron diffraction pattern obtained for **1**, dosed with 0.3 equivalents of D_2 per pore (λ = 2.0772 Å; T = 9 K). Black circles show the experimental diffraction pattern and the red line represents the calculated diffraction pattern. The gray line represents the difference between observed and calculated patterns, the blue tick marks indicate calculated Bragg peak positions of the activated phase **1**, the purple tick marks indicate calculated Bragg peak positions of the D_2 adsorbed phase **1–D₂**, and the pink tick marks indicate reflections from the aluminum sachet.

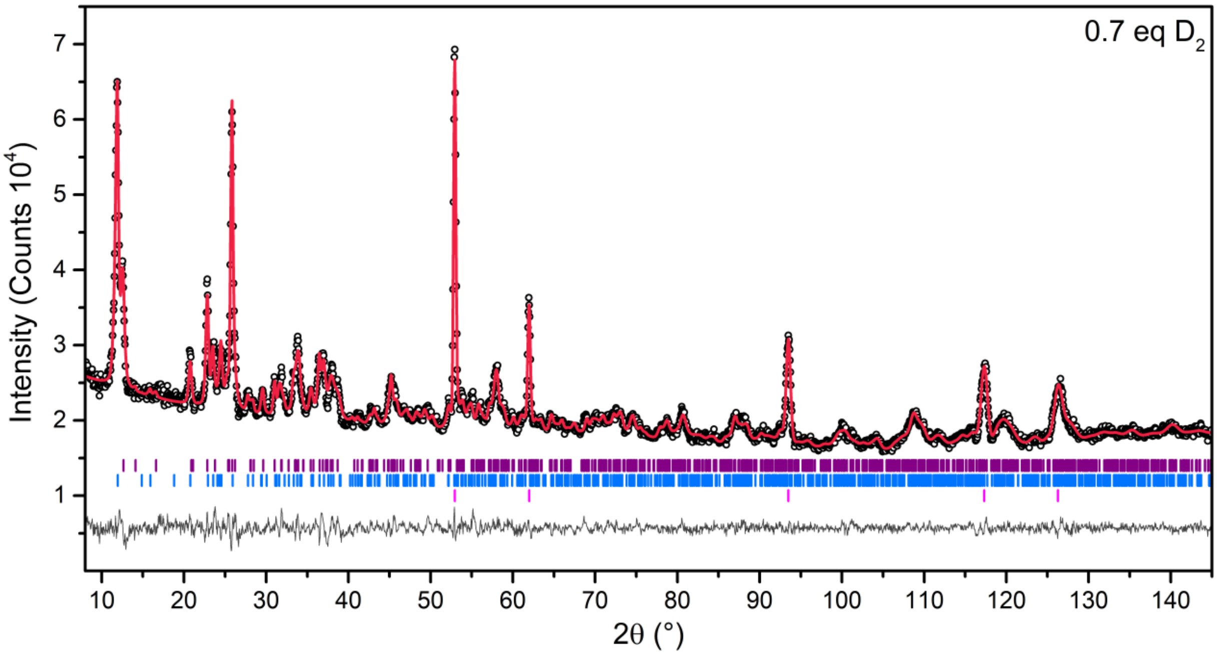

Figure S11. Rietveld refinement of the powder neutron diffraction pattern obtained for **1**, dosed with 0.7 equivalents of D_2 per pore (λ = 2.0772 Å; T = 9 K). Black circles show the experimental diffraction pattern and the red line represents the calculated diffraction pattern. The gray line represents the difference between observed and calculated patterns, the blue tick marks indicate calculated Bragg peak positions of the activated phase **1**, the purple tick marks indicate calculated Bragg peak positions of the D_2 adsorbed phase **1–D$_2$**, and the pink tick marks indicate reflections from the aluminum sachet.

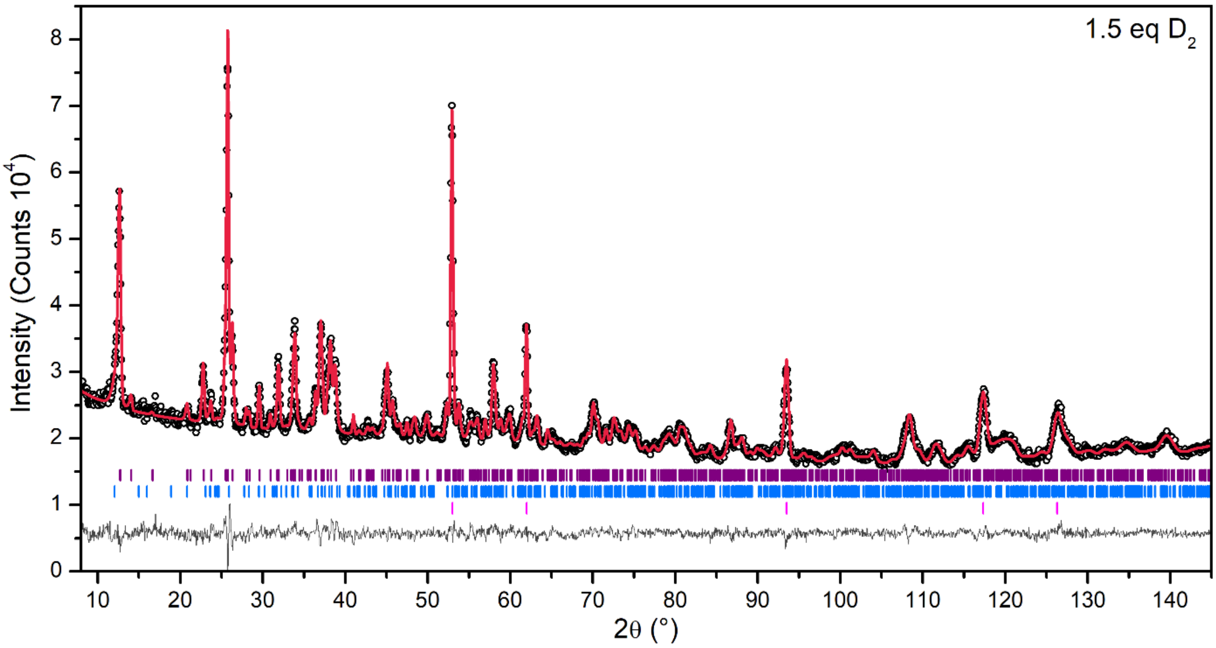

Figure S12. Rietveld refinement of the powder neutron diffraction pattern obtained for **1**, dosed with 1.5 equivalents of D_2 per pore (λ = 2.0772 Å; T = 9 K). Black circles show the experimental diffraction pattern and the red line represents the calculated diffraction pattern. The gray line represents the difference between observed and calculated patterns, the blue tick marks indicate calculated Bragg peak positions of the activated phase **1**, the purple tick marks indicate calculated Bragg peak positions of the D_2 adsorbed phase **1–D$_2$**, and the pink tick marks indicate reflections from the aluminum sachet.

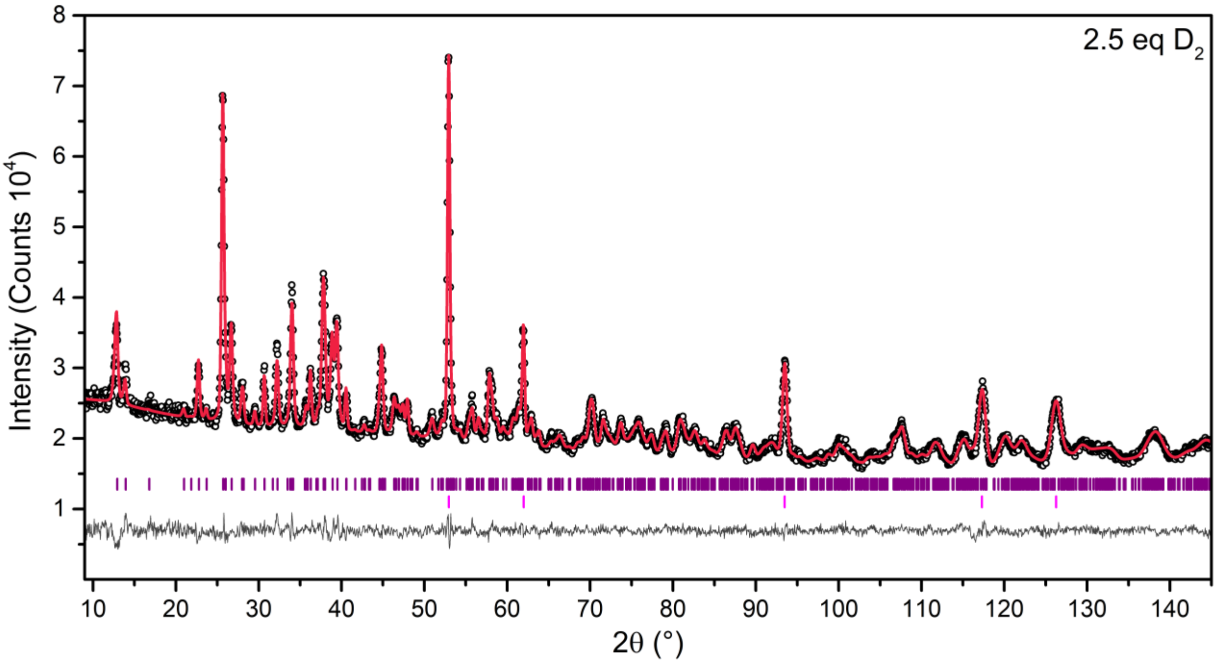

Figure S13. Rietveld refinement of the powder neutron diffraction pattern obtained for **1**, dosed with 2.5 equivalents of D_2 per pore (λ = 2.0772 Å; T = 9 K). Black circles show the experimental diffraction pattern and the red line represents the calculated diffraction pattern. The gray line represents the difference between observed and calculated patterns, the purple tick marks indicate calculated Bragg peak positions of the D_2 adsorbed phase **1–D_2**, and the pink tick marks indicate reflections from the aluminum sachet.

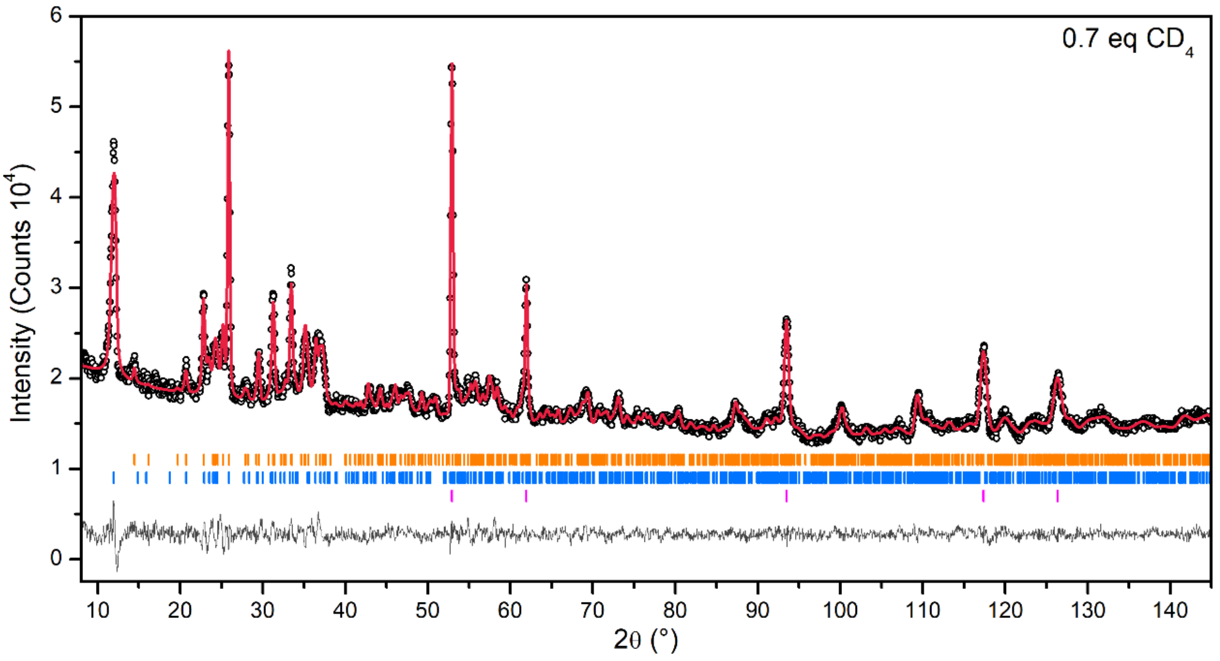

Figure S14. Rietveld refinement of the powder neutron diffraction pattern obtained for **1**, dosed with 0.7 equivalents of CD_4 per pore (λ = 2.0772 Å; T = 7 K). Black circles show the experimental diffraction pattern and the red line represents the calculated diffraction pattern. The gray line represents the difference between observed and calculated patterns, the blue tick marks indicate calculated Bragg peak positions of the activated phase **1**, the orange tick marks indicate calculated Bragg peak positions of the CD_4 adsorbed phase **1–CD_4**, and the pink tick marks indicate reflections from the aluminum sachet.

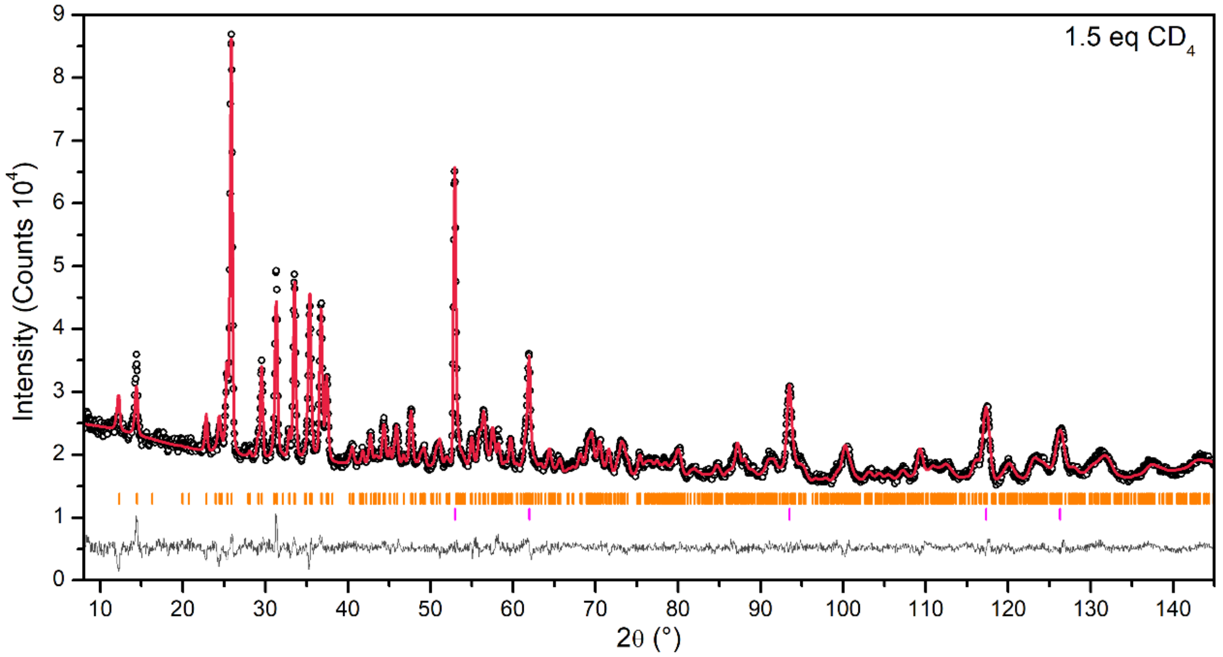

Figure S15. Rietveld refinement of the powder neutron diffraction pattern obtained for **1**, dosed with 1.5 equivalents of CD_4 per pore (λ = 2.0772 Å; T = 7 K). Black circles show the experimental diffraction pattern and the red line represents the calculated diffraction pattern. The gray line represents the difference between observed and calculated patterns, the orange tick marks indicate calculated Bragg peak positions of the CD_4 adsorbed phase **1–CD₄**, and the pink tick marks indicate reflections from the aluminum sachet.

Table S3. Figures of merit and unit cell parameters (space group *C2/c*) obtained from Rietveld refinements to the neutron powder diffraction data measured at 9 K (**1**, and **1–D₂**) or 7 K (**1–CD₄**) as presented in figures S9 – S15. Values in parenthesis indicate one standard deviation.

	R_{wp}	R_p	R_{exp}	GoF	X^2	*a* (Å)	*b* (Å)	*c* (Å)	α (°)	β (°)	γ (°)	*V* (Å^3)
***1**, activated*	0.0228	0.0186	0.0203	1.14	1.265	17.587(1)	12.812(1)	9.2999(5)	90	114.814(4)	90	1902.1(2)
1 + 0.3 *eq.* D_2	0.0245	0.0200	0.0216	1.13	1.280	18.361(7)	11.35(1)	9.335(2)	90	113.64(3)	90	1782(2)
1 + 0.7 *eq.* D_2	0.0252	0.0207	0.0221	1.16	1.283	18.402(3)	11.324(5)	9.333(1)	90	113.659(8)	90	1781.4(8)
1 + 1.5 *eq.* D_2	0.0242	0.0195	0.0215	1.14	1.268	18.456(1)	11.233(1)	9.3508(4)	90	113.648(3)	90	1775.8(2)
1 + 2.5 *eq.* D_2	0.0223	0.0185	0.0214	1.06	1.088	18.665(1)	10.9486(8)	9.3838(5)	90	113.807(3)	90	1754.5(2)
1 + 0.7 *eq.* CD_4	0.0274	0.0224	0.0240	1.17	1.301	17.968(2)	12.098(2)	9.3121(6)	90	114.006(4)	90	1849.1(4)
1 + 1.5 *eq.* CD_4	0.0260	0.0212	0.0222	1.18	1.358	18.031(1)	11.9665(7)	9.3206(4)	90	113.856(3)	90	1839.3(2)

7. Single crystal X-ray diffraction

Single crystals of **1–H_2O** were coated in paratone oil under air prior to transport to the Advanced Light Source at Lawrence Berkeley National Lab (station 12.2.1). Crystals were evaluated by polarized light microscopy and mounted on a MiTeGen 10 μm aperture Dual-Thickness MicroMount. X-ray diffraction data were collected with a silicon monochromated synchrotron radiation beam of 17 keV (λ = 0.7288 Å). The crystal was cooled to 100 K with a dry nitrogen stream. Bruker APEX3 software was used for the data collections,[14] Bruker SAINT V8.38A software was used to conduct the cell refinement and data reduction procedures.[15] Absorption corrections were carried out by a multi-scan method utilizing the SADABS program.[16] The initial structure solution was found using direct methods (SHELXS), and refinements were carried out using SHELXL-2014.[17–19] Thermal parameters for all non-hydrogen atoms were refined anisotropically and all framework hydrogens were refined using the riding model. The pore water molecules in **1–H_2O** were disordered and could not be modeled with high precision; however, the pore water oxygens (four per MOF pore, in agreement with TGA analysis) were located from the Fourier difference map and were allowed to refine freely without restraints or constraints and without hydrogen atoms. The structure of **1–H_2O** has been deposited to the Cambridge Crystallographic Data Centre (CCDC), with deposition number 1996337.

Table S4. Single crystal X-ray diffraction data and structure refinement details for **1–H_2O**.

parameter	**1–H_2O**
Formula	$C_{16}H_8O_{12}U$
Formula weight	630.25
Temperature (K)	100
Crystal size (mm^3)	0.035 × 0.005 × 0.005
Crystal system	monoclinic
Space group	*C*2/*c*
a, *b*, *c* (Å)	17.7447(17), 12.5982(12), 9.2934(8)
α, β, γ(°)	90, 114.702(4), 90
V, (Å^3)	1887.4(3)
Z / Z″	8 / 4
Radiation, λ (Å)	0.7288
2Θ Range for data collection (°)	5.182 to 54.19
Completeness (%) to 2Θ = 51.86 °	99.9
Data / Restraints / Parameters	1944 / 0 / 133
Goodness of fit on F^2	1.087
R_1^a, wR_2^b ($I > 2\sigma$ (I))	0.0531, 0.1493
R_1^a, wR_2^b (all data)	0.0667, 0.1605
Peak and hole (*e* Å^{-3})	2.33, −1.95

$^a\ R_1 = \frac{\sum\left||F_o| - |F_c|\right|}{\sum|F_o|}$ $^b\ wR_2 = \left\{\frac{\sum\left[w\left(F_o^2 - F_c^2\right)^2\right]}{\sum[w(F_o^2)^2]}\right\}^{\frac{1}{2}}$

Table S5. Selected bond distances in Å and angles in degrees for **1–H_2O**. Angles φ_1 and φ_2 describe the U···U···U angles of the idealized parallelogram spanned by the four corner U atoms within the crystallographic *ab*-plane of each pore, as illustrated in Figure 5a of the main text. Angle φ_1 is reported as the average between the two angles φ_{1a} and φ_{1b}, which slightly deviate from one another due to a three-dimensional distortion of the idealized parallelogram. Angles ω_1 and ω_2 describe the dihedral angle between O–U···U–O planes and O–C–O planes at each side of a bdc^{2-} linker, as illustrated in Figure 5b of the main text. Values in parenthesis indicate one standard deviation.

	U–O (Å) [a]	∠O–U–O(°) [b]	φ_1 (°)	φ_2 (°)	ω_1 (°)	ω_2 (°)
**1–H_2O**	2.398(7)<br>2.312(8)<br>2.300(7)<br>2.377(7)	73.1(3)<br>71.9(3)<br>75.5(3)<br>72.4(3)<br>115.1(3)<br>114.8(3)	75.639(3)	104.361(1)	154.4(9)	174.7(9)

[a] The two squares formed by the eight oxygen atoms coordinated to each U ion in square anti-prismatic geometry are related by symmetry, resulting in a total of four independent U–O bonds.

[b] The two squares formed by the eight oxygen atoms coordinated to each U ion in square anti-prismatic geometry are related by symmetry, resulting in six independent O–U–O angles that can be constructed between all oxygen atoms of one plane and the uranium ion.

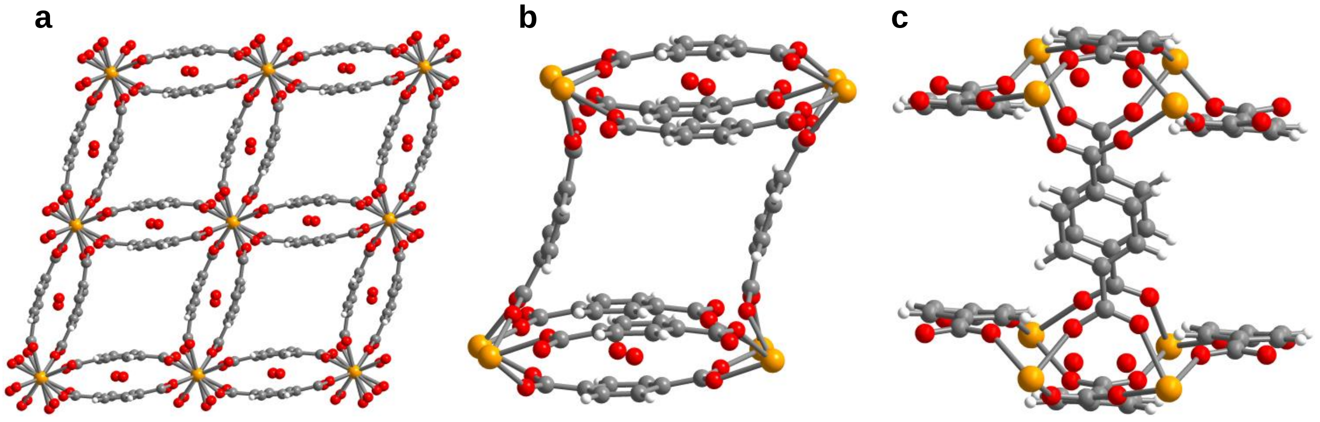

Figure S16. (a) Graphical representation of single crystal structure data obtained for **1–H_2O**, including the refined oxygen atoms of H_2O molecules adsorbed in the pores, shown in a larger fraction of the structural with multiple pores. (b) Graphical representation of one pore with viewing direction along the crystallographic *c*-axis. (c) Graphical representation of the same pore as in (b), but rotated by 90°. Orange, red, grey, and white spheres represent U, O, C, and H atoms, respectively.

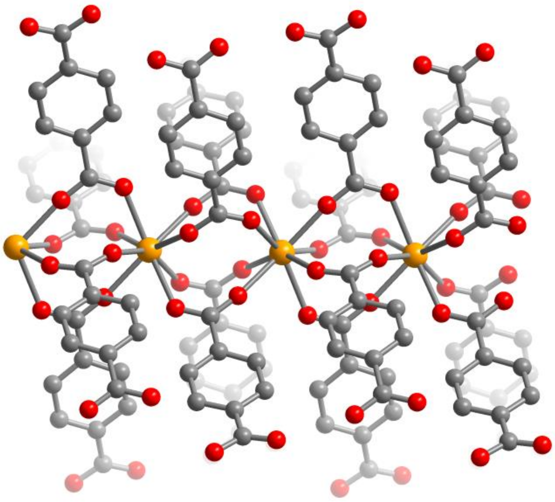

Figure S17. Graphical representation of single crystal structure data obtained for **1–H_2O**, truncated to show the uranium chains that propagate along the crystallographic *c*-axis of **1–H_2O**. Orange, red, and grey spheres represent U, O, and C atoms, respectively.

8. References

1 M. J. Monreal, R. K. Thomson, T. Cantat, N. E. Travia, B. L. Scott and J. L. Kiplinger, *Organometallics*, 2011, **30**, 2031.

2 J. A. Mason, J. Oktawiec, M. K. Taylor, M. R. Hudson, J. Rodriguez, J. E. Bachman, M. I. Gonzalez, A. Cervellino, A. Guagliardi, C. M. Brown, P. L. Llewellyn, N. Masciocchi and J. R. Long, *Nature*, 2015, **527**, 357.

3 A. C. Larson, R.B. von Dreele, "General Structure Analysis System (GSAS)" Los Alamos National Laboratory Report LAUR, 2000, 86.

4 C. Falaise, A. Assen, I. Mihalcea, C. Volkringer, A. Mesbah, N. Dacheux and T. Loiseau, *Dalton Trans.*, 2015, **44**, 2639.

5 D. E. Jaramillo, D. A. Reed, H. Z. H. Jiang, J. Oktawiec, M. W. Mara, A. C. Forse, D. J. Lussier, R. A. Murphy, M. Cunningham, V. Colombo, D. K. Shuh, J. A. Reimer and J. R. Long, *Nat. Mater.*, 2020, **19**, 517.

6 A. Coelho and Topas Academic v6 Coelho Software, 2017, http://www.topas-academic.net/.

7 Y. G. Andreev, G. S. MacGlashan and P. G. Bruce, *Phys. Rev. B*, 1997, **55**, 12011.

8 B. H. Toby, *J. Appl. Cryst.*, 2001, **34**, 210.

9 G. S. Pawley, *J. Appl. Cryst.*, 1981, **14**, 357.

10 H. M. Rietveld, *J. Appl. Cryst.*, 1969, **2**, 65.

11 T. Runčevski, M. T. Kapelewski, R. M. Torres-Gavosto, J. D. Tarver, C. M. Brown and J. R. Long, *Chem. Commun.*, 2016, **52**, 8251.

12 R. A. Pollock, J.-H. Her, C. M. Brown, Y. Liu and A. Dailly, *J. Phys. Chem. C*, 2014, **118**, 18197.

13 H. Wu, W. Zhou and T. Yildirim, *J. Am. Chem. Soc.*, 2007, **129**, 5314.

14 APEX3 Version 2016.1-0 ed., Bruker AXS, Inc.: Madison, WI, USA, 2016.

15 SAINT Version 8.38A ed., Bruker AXS, Inc.: Madison, WI, USA.

16 Sheldrick, George M., Bruker AXS, Inc.: Madison, WI, USA, SADABS, Version 2014/4, 2014.

17 G. M. Sheldrick, *Acta. Cryst. A*, 2008, **64**, 112.

18 O. V. Dolomanov, L. J. Bourhis, R. J. Gildea, J. A. K. Howard and H. Puschmann, *J. Appl. Cryst.*, 2009, **42**, 339.

19 L. J. Farrugia, *J. Appl. Cryst.*, 2012, **45**, 849.
